# Elevated triglyceride glucose-body mass index is associated with a higher risk of reduced cumulative live birth and adverse pregnancy outcomes in women undergoing assisted reproductive technology: a retrospective cohort study

**DOI:** 10.3389/fendo.2026.1842023

**Published:** 2026-06-17

**Authors:** Jie Hu, Shujuan Ma, Yinyan Gao, Yangqin Peng, Xiaojuan Wang, Irene X. Y. Wu, Fei Gong

**Affiliations:** 1 Xiangya School of Public Health, Central South University, Changsha, Hunan, China; 2 Clinical Research Center for Reproduction and Genetics in Hunan Province, Reproductive and Genetic Hospital of CITIC-Xiangya, Changsha, Hunan, China; 3 NHC Key Laboratory of Human Stem Cell and Reproductive Engineering, Xiangya School of Basic Medical Sciences, Central South University, Changsha, Hunan, China; 4 Central South University, Hunan Provincial Key Laboratory of Clinical Epidemiology, Changsha, Hunan, China

**Keywords:** adverse pregnancy outcomes, assisted pregnancy outcomes, assisted reproductive technology, cumulative live birth rate, Infertility, triglyceride glucose-body mass index

## Abstract

**Background:**

Insulin resistance has been linked to adverse reproductive and pregnancy outcomes. The triglyceride glucose–body mass index (TyG-BMI) serves as a simple and cost-effective surrogate marker of insulin resistance. This study aimed to investigate the association between maternal TyG-BMI and cumulative live birth as well as pregnancy outcomes in women undergoing assisted reproductive technology.

**Methods:**

This retrospective cohort study included 53,393 women undergoing assisted reproductive technology at the Reproductive and Genetic Hospital of CITIC-Xiangya, China, between January 1, 2017, and March 31, 2023. Associations between baseline TyG-BMI and assisted pregnancy outcomes were estimated using modified Poisson regression and restricted cubic splines.

**Results:**

A significant linear inverse association was observed between TyG-BMI and cumulative live birth rate (*P*-overall = 0.029). Compared with women in the lowest TyG-BMI tertile (≤177), those in the highest tertile (>203) had a lower cumulative live birth rate (aRR 0.973; 95% confidence interval 0.956–0.991). Higher TyG-BMI was also associated with higher risks of first-trimester miscarriage (aRR, 1.090; 95% confidence interval 1.002-1.185), second- or third-trimester fetal loss (aRR 1.694; 95% confidence interval 1.481–1.938), preterm birth (aRR 1.244; 95% confidence interval 1.170–1.323), and macrosomia (aRR 2.352; 95% confidence interval 2.060–2.685).

**Conclusions:**

Higher maternal TyG-BMI is associated with reduced cumulative live birth rates and higher risks of adverse pregnancy outcomes including miscarriage, preterm birth, and macrosomia following assisted reproductive technology. TyG-BMI may be a potential and cost-effective marker associated with adverse reproductive outcomes in women undergoing fertility treatment.

**Clinical Trial Registration:**

https://www.clinicaltrials.gov, identifier NCT05404464.

## Introduction

Infertility is defined as the inability to achieve a clinical pregnancy after 12 or more months of unprotected intercourse (1). According to the World Health Organization, approximately one in six adults worldwide are affected by infertility, making it a significant public health concern. Assisted reproductive technologies (ART), particularly *in vitro* fertilization (IVF) and intracytoplasmic sperm injection (ICSI), have emerged as effective treatments for infertility (2). However, success rates of ART vary across countries, with live birth rates ranging from 25.0% to 41.5% (3). Key predictors of ART success include age, ovarian reserve, medical history, and metabolic factors (4).

Metabolic disorders, including insulin resistance and dyslipidemia, play a crucial role in both infertility development and adverse assisted pregnancy outcomes, such as macrosomia and premature birth (5–8). The triglyceride glucose (TyG) index, calculated from fasting plasma glucose (FPG) and fasting triglycerides (TG), has been proposed as a feasible and cost-effective surrogate for insulin resistance assessment (9, 10). Recent studies in the general population have linked elevated TyG levels with adverse pregnancy outcomes, such as gestational diabetes, gestational hypertension, macrosomia, large-for-gestational-age infants, and preterm birth (11–14).

Given that obesity is an established driver of insulin resistance (15), the TyG-BMI—the TyG index augmented by the body mass index (BMI)—has been proposed for early identification of insulin resistance (16). The added value of TyG-BMI as a composite indicator has already been established in prior studies, which consistently demonstrated that TyG-BMI may outperform TyG alone in assessing insulin resistance and predicting cardiometabolic risk (17–19). However, the relationship between TyG-BMI and reproductive outcomes remains unclear, particularly among individuals with high-fertility demands undergoing ART.

To date, only three studies have examined the association between TyG-BMI and assisted reproductive outcomes. Two exclusively focused on patients with polycystic ovary syndrome (20, 21), whereas the remaining one study was specifically conducted among the *in vitro* fertilization and embryo transfer (IVF-ET) population (22). These studies were conducted in populations with specific metabolic disturbances (such as PCOS) or under restricted treatment modalities (such as IVF-ET) and primarily evaluated reproductive outcomes from individual embryo transfer cycles, rather than reflecting the overall ART process, which may limit the generalizability and clinical interpretability of their findings. Importantly, whether these associations extend to a broader, unselected ART population remains unclear. In addition, the cumulative live birth rate (CLBR), a more clinically meaningful and patient-centered endpoint reflecting the overall success of ART, has not been adequately examined in relation to TyG-BMI.

Therefore, building upon an existing large-scale cohort, this study aimed to further extend the clinical relevance of TyG-BMI from CLBR to adverse pregnancy outcomes including preterm birth and macrosomia, providing more comprehensive evidence for the associations between TyG-BMI and reproductive outcomes in the overall ART population. As a simple and low-cost indicator, TyG-BMI may be associated with adverse reproductive outcomes and may provide additional information for assessing the metabolic status for individuals undergoing ART.

## Methods

This study was approved by the Ethics Committee of the Reproductive and Genetic Hospital of CITIC-Xiangya, Changsha, People’s Republic of China (approval number, LL-SC-2025-019), and followed the Strengthening the Reporting of Observational Studies in Epidemiology (STROBE) reporting guideline (23).

### Study design, setting, and participants

**Figure 1:**
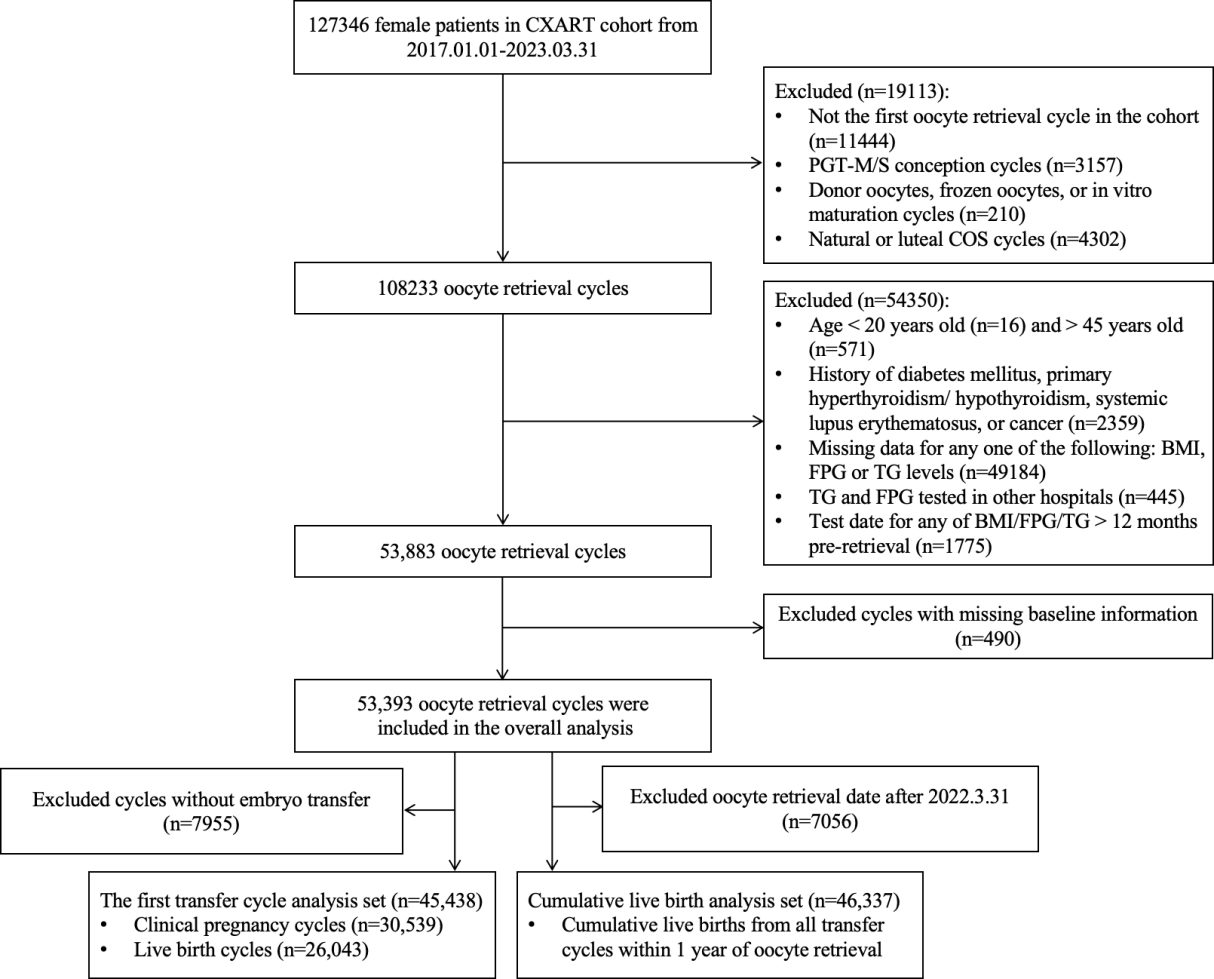
Flowchart of the participants selection. Participants were excluded in three stages: (1) based on predefined inclusion and exclusion criteria (n = 73,463); (2) missing key variables (n = 490); and (3) analysis-specific exclusions, including without embryo transfer in the first transfer cycle dataset (n = 7,995) and less than 1 year since oocyte retrieval for the cumulative live birth analysis (n = 7,056). PGT-M, Preimplantation Genetic Testing for Monogenic Disorders; PGT-S, Preimplantation Genetic Testing for Structural Rearrangements; COS, controlled ovarian stimulation; BMI, body mass index; FPG, fasting plasma glucose; TG, triglyceride.

This retrospective cohort study was conducted based on the CITIC-Xiangya Assisted Reproductive Technology (CXART) Cohort, an ongoing longitudinal and prospective study located in southern China that encompasses comprehensive health data from 309,171 ART cycles involving 157,398 infertile couples and an extensive collection of 157,990 biomaterials, with the primary objective of establishing a substantial data resource in the realm of human reproduction (24). This study included women who underwent ART at the Reproductive and Genetic Hospital of CITIC-Xiangya in China from January 1, 2017, to March 31, 2023. The inclusion criteria were as follows: (1) the first oocyte retrieval cycle in the CXART cohort; (2) ≥20 years old and ≤45 years old; (3) data on TG, FPG, and BMI were available; (4) test date for any of BMI/FPG/TG ≤12 months pre-retrieval. The exclusion criteria were as follows: (1) PGT-M/S conception cycles; (2) with history of diabetes mellitus, primary hyperthyroidism/hypothyroidism, systemic lupus erythematosus, or cancer; (3) missing data for any one of the following: BMI, FPG, or TG; (4) cycles with donor oocytes, frozen oocytes, or *in vitro* maturation; (5) natural and luteal controlled ovarian stimulation cycles. Natural cycles were excluded as natural oocyte retrievals were seldom followed by embryo culture or transfer, and luteal phase stimulation cycles were excluded as some were recorded as subsequent retrieval cycles and therefore not consistently captured within the initial cycle dataset. Ultimately, 45,438 participants were included in the first transfer cycle analysis set, and 46,337 participants were included in the cumulative live birth analysis set (**Figure 1**).

### Ascertainment of exposure

The TyG-BMI was calculated as the natural logarithm of [TG (mg/dL)×FPG (mg/dL)/2]×BMI (16). Fasting blood samples were collected from all participants after an overnight fast of at least 8 h prior to the initiation of ART treatment. FPG was measured using the hexokinase method, and TG were determined using an enzymatic oxidase method. All biochemical analyses were performed on a Roche cobas automated analyzer (Roche Diagnostics GmbH, Mannheim, Germany) using commercially available assay kits from the same manufacturer. BMI was calculated based on anthropometric measurements of height and weight. All blood sample collection and laboratory analyses were conducted by trained professionals in a tertiary hospital in accordance with standard operating procedures (SOPs) to ensure data quality and consistency. A 12-month window was adopted to capture the most recent metabolic measurements before oocyte retrieval, consistent with routine clinical data validity and to maximize data utilization.

### Ascertainment of outcomes

The primary outcome was the cumulative live birth rate (CLBR), which was defined as the probability of achieving live birth arising from fresh or frozen-thawed embryo transfers from the study cycle within 12 months after oocyte retrieval. The secondary outcomes were pregnancy and prenatal outcomes in the first transfer cycle, including clinical pregnancy (defined as one or more gestational sacs with fetal heart activity under ultrasonography 4 weeks after embryo transfer), live birth (defined as one or more living infants of any gestational age, multiple births counted as one live birth delivery), first-trimester miscarriage (defined as intrauterine pregnancy loss after confirmation of gestational sacs during the first trimester), second/third-trimester fetal loss (defined as the loss of an intrauterine pregnancy during the second or third trimester), macrosomia (defined as singleton pregnancy and with birth weight ≥4,000 g), and preterm birth (<37 weeks of gestation).

### Ascertainment of covariates

**Figure 2:**
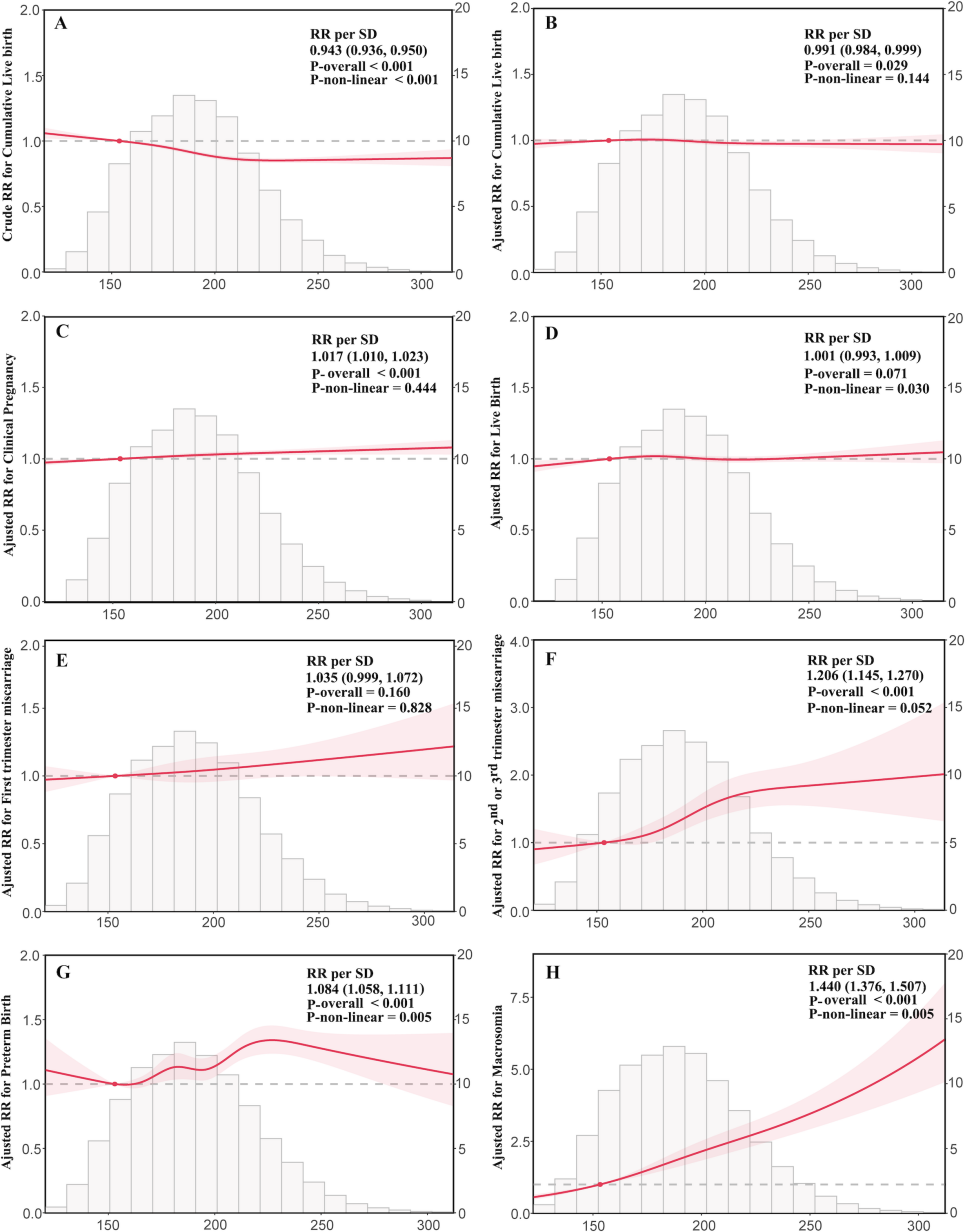
Restricted cubic spline curves of the association between TyG-BMI and assisted reproductive outcomes. The figure illustrates the crude RR for cumulative live birth **(A)**, adjusted RRs for cumulative live birth **(B)**, clinical pregnancy **(C)**, live birth **(D)**, first-trimester miscarriage **(E)**, second- or third-trimester fetal loss **(F)**, preterm birth **(G)**, and macrosomia **(H)** relative to TyG-BMI levels. Adjusted RRs (depicted as red lines) with their 95% confidence intervals (shown as pink shading) were based on restricted cubic spline models. These models assessed TyG-BMI on continuous scales, using the 10th percentile as reference points. The model was adjusted for female age, education level, smoking, alcoholism, hypothyroidism, polycystic ovary syndrome, anti-Müllerian hormone, pre-pregnancy hypertension, pre-pregnancy diabetes, gravidity, and parity. TyG-BMI, triglyceride glucose-body mass index; RR, risk ratio; SD, standard deviation.

To visualize the potential causal association between TyG-BMI and pregnancy outcomes (CLRB, clinical pregnancy, live birth, and miscarriage), as well as neonatal outcomes (preterm birth and macrosomia), directed acyclic graphs (DAG) were generated using the online tool DAGitty v3.0 (http://www.dagitty.net/dags.html#), informed by existing literature and clinical knowledge. Covariates adjusted in the final models were identified *a priori* as confounders based on DAGs, including demographic information (female age, education level, current smoking status, and alcohol consumption), reproductive history (parity and gravidity), infertility assessment (polycystic ovary syndrome), medical history (clinical or subclinical hypothyroidism, diabetes, and hypertension), and clinical examination and detection (anti-Müllerian hormone). The demographic information, reproductive history, and medical history were derived from outpatient records. The infertility assessment was confirmed through medical records. The corresponding DAGs are presented in **Supplementary Figure 1** and **Figure 2**.

### Statistical analysis

To comprehensively evaluate the association between TyG-BMI and reproductive outcomes across different stages of ART treatment, we conducted three complementary analyses with distinct analytical scopes. First, the primary analysis was performed at the level of the oocyte retrieval cycle, using cumulative live birth within 1 year as the primary outcome, based on the cumulative live birth analysis set. Each initiated oocyte retrieval cycle was followed for up to 1 year, and all derived embryo transfers were considered. Cycles that did not result in transferable embryos, canceled transfers, or failed transfers were classified as not achieving cumulative live birth. This approach captures the entire treatment pathway from ovarian stimulation to live birth and avoids conditioning on intermediate events. Second, the secondary analysis was performed at the level of the first transfer cycle, based on the first transfer cycle analysis set. Analyses of all pregnancy outcomes (including live birth, clinical pregnancy, miscarriage, preterm birth, and macrosomia) were restricted to women who underwent embryo transfer. These analyses were intended to provide stage-specific insights into post-transfer outcomes. Third, given that TyG-BMI may influence the likelihood of embryo transfer, we also evaluated its association with the probability of reaching embryo transfer to avoid selection bias.

Participants were divided into three groups according to tertiles of the TyG-BMI in the overall analysis dataset (tertile 1 (T1): ≤177, tertile 2 (T2): (177-203], tertile 3 (T3): >203). Participant demographics, baseline clinical characteristics, embryo transfer, and pregnancy outcomes were described across tertiles of the TyG-BMI. Mean ± standard deviation or median (interquartile range) were used to describe continuous data, depending on distribution, whereas frequencies and percentages were used to describe categorical data. The normality of continuous variables was verified using the Anderson–Darling test. Differences among groups were determined using the one-way ANOVA, Kruskal–Wallis test, or Pearson chi-square test, as appropriate.

Multivariate modified Poisson regression was used to estimate the relative risks (RRs), absolute risks (ARs), and 95% confidence intervals (CIs) of different reproductive outcomes associated with the TyG-BMI. Confounding factors were identified based DGAs and adjusted in models for each outcome.

Restricted cubic spline (RCS) regression models were employed to explore the dose–response relationships between TyG-BMI and reproductive outcomes. Confounding factors were adjusted for each outcome in the results of RCS. The TyG-BMI values were included in the analyses as continuous variables, with their distributions displayed in histograms. The 10th percentiles were assigned as the reference values (RR = 1.00) (25). The RCS models were optimized for accuracy and overfitting using the Akaike information criterion (AIC). The knots were determined based on the minimum AIC value. The knots of clinical pregnancy, first-trimester miscarriage, and macrosomia were set at the 10th, 50th, and 90th percentiles of the TyG-BMI values, whereas cumulative live birth, live birth, second- or third-trimester fetal loss and preterm birth with knots at the 5th, 35th, 65th, and 95th percentiles of the TyG-BMI values.

Prespecified subgroup analyses were conducted to evaluate potential effect modification. Subgroup variables were selected *a priori* based on previous literature and clinically relevant factors commonly considered in ART, including demographic characteristics, infertility-related conditions, and treatment-related factors (female age, smoking, sterility type, clinical or subclinical hypothyroidism, polycystic ovary syndrome, untreated hydrosalpinx, uterine adhesions, adenomyosis, leiomyoma, endometriosis, male factor, metformin and pioglitazone diabetes drug usage, and ART methods). Effect modification was assessed using formal tests for interaction, with *P* interaction calculated from the likelihood ratio test comparing with and without the multiplicative term. Subgroup interpretations were based on these interaction tests. To account for multiple comparisons, Benjamini and Hochberg multiple comparison adjustments were applied in subgroup analysis. In addition, sensitivity analyses were conducted by restricting BMI/FPG/TG measurements to narrower time windows (≤3 and ≤6 months prior to oocyte retrieval) to assess potential exposure misclassification.

Due to the completeness of the baseline data exceeding 98% (**Supplementary Table 1**), this study was conducted based on the complete dataset and no multiple imputation was performed. All statistical tests were two-sided, with *P* < 0.05 considered significant. Analyses were conducted using R version 4.5.1.

## Results

### Baseline characteristics of study participants

Baseline characteristics across TyG-BMI tertiles in the cumulative live birth analysis set are shown in **Table 1**. Among 46,337 participants in the cumulative live birth analysis set (mean age 31.0 years), 15,702 (33.9%) were of low TyG-BMI, 15,168 (32.7%) were of medium TyG-BMI, and 15,467 (33.4%) were of high TyG-BMI. Older age, higher BMI, more parity and gravidity, and more common conditions such as secondary infertility, current smoking, hypertension, diabetes, clinical or subclinical hypothyroidism, adenomyosis, leiomyoma, and untreated hydrosalpinx were observed in T2-T3 groups, in contrast to the T1 group. T2-T3 groups also tended to have higher levels of systolic blood pressure, diastolic blood pressure, total cholesterol, and low-density lipoprotein cholesterol and lower levels of high-density lipoprotein cholesterol, anti-Müllerian hormone, and antral follicle count compared with the T1 group. *In vitro* fertilization (IVF) was the predominant ART methods (67.8%), whereas the agonist protocol was the most common stimulation protocol (75.0%). No significant difference regarding polycystic ovary syndrome was observed among three groups (*P* > 0.05).

**Table 1:** Baseline characteristics of participants by tertiles of TyG-BMI in cumulative live birth analysis set.

Characteristics	Total (n=46,337)	Tertile 1 (n=15,702)	Tertile 2 (n=15,168)	Tertile3 (n=15,467)	P
Male age (years)	32.9 (30.0, 37.1)	31.9 (29.3, 35.3)	33.1 (30.1, 37.4)	33.9 (30.7, 38.5)	<0.001
Maternal variables					
Female age (years)	31.0 (28.0, 34.0)	30.0 (27.0, 33.0)	31.0 (28.0, 34.0)	32.0 (28.0, 36.0)	<0.001
BMI (kg/m2)	22.5 (20.6, 24.3)	19.8 (18.8, 20.8)	22.6 (21.8, 23.4)	25.0 (24.0, 26.4)	<0.001
Age at menarche (years)	13.0 (13.0, 14.0)	13.0 (13.0, 14.0)	13.0 (13.0, 14.0)	13.0 (13.0, 14.0)	<0.001
Education					<0.001
Primary school or below	21,929 (47.3)	6,330 (40.3)	7,147 (47.1)	8,452 (54.6)	
High school	7,221 (15.6)	2,542 (16.2)	2,388 (15.7)	2,291 (14.8)	
University or above	17,187 (37.1)	6,830 (43.5)	5,633 (37.1)	4,724 (30.5)	
Infertility years	3.0 (2.0, 5.0)	3.0 (2.0, 4.0)	3.0 (2.0, 5.0)	3.0 (2.0, 5.0)	<0.001
Infertility type					<0.001
Primary	19,246 (41.5)	7,021 (44.7)	5,982 (39.4)	6,243 (40.4)	
Secondary	27,091 (58.5)	8,681 (55.3)	9,186 (60.6)	9,224 (59.6)	
Female current smoking	881 (1.9)	246 (1.6)	256 (1.7)	379 (2.5)	<0.001
Female alcoholism	66 (0.1)	23 (0.1)	24 (0.1)	19 (0.1)	0.704
Hypertension	722 (1.6)	69 (0.4)	191 (1.3)	462 (3.0)	<0.001
Diabetes	159 (0.3)	14 (0.1)	36 (0.2)	109 (0.7)	<0.001
Clinical/subclinical hypothyroidism	1,613 (3.5)	440 (2.8)	507 (3.3)	666 (4.3)	<0.001
Gravidity					<0.001
0	19,129 (41.3)	6,981 (44.5)	5,945 (39.2)	6,203 (40.1)	
1	11,354 (24.5)	3,881 (24.7)	3,662 (24.1)	3,811 (24.6)	
≥2	15,854 (34.2)	4,840 (30.8)	5,561 (36.7)	5,453 (35.3)	
Parity					<0.001
0	35,978 (77.6)	13,107 (83.5)	11,565 (76.2)	11,306 (73.0)	
1	8,953 (19.3)	2,321 (14.8)	3,144 (20.7)	3,488 (22.6)	
≥2	1,406 (3.1)	274 (1.7)	459 (3.0)	673 (4.4)	
Infertility related diseases					
PCOS	14,421 (31.1)	4,918 (31.3)	4,628 (30.5)	4,875 (31.5)	0.131
Male factor	13,487 (29.1)	4,578 (29.2)	4,289 (28.3)	4,620 (29.9)	0.009
Uterine adhesions	8,464 (18.3)	3,120 (19.9)	2,877 (19.0)	2,467 (16.0)	<0.001
Adenomyosis	2,097 (4.5)	546 (3.5)	687 (4.5)	864 (5.6)	<0.001
Leiomyoma	8,114 (17.5)	2,175 (13.9)	2,800 (18.5)	3,139 (20.3)	<0.001
Endometriosis	3,814 (8.2)	1,280 (8.2)	1,227 (8.1)	1,307 (8.5)	0.468
Untreated hydrosalpinx	4,731 (10.2)	1,463 (9.3)	1,541 (10.2)	1,727 (11.2)	<0.001
Chocolate cyst	975 (2.1)	414 (2.6)	316 (2.1)	245 (1.6)	<0.001
Recurrent miscarriage	2,826 (6.1)	1,113 (7.1)	1,010 (6.7)	703 (4.5)	<0.001
Clinical data at baseline					
SBP (mmHg)	115.0 (108.0, 122.0)	112.0 (105.0, 119.0)	115.0 (108.0, 122.0)	118.0 (111.0, 126.0)	<0.001
DBP (mmHg)	75.0 (69.0, 81.0)	73.0 (68.0, 79.0)	75.0 (70.0, 81.0)	77.0 (71.0, 84.0)	<0.001
TC (mmol/L)	4.3 (3.9, 4.8)	4.2 (3.8, 4.7)	4.3 (3.9, 4.8)	4.5 (4.0, 5.0)	<0.001
HDL-C (mmol/L)	1.4 (1.2, 1.6)	1.6 (1.4, 1.8)	1.4 (1.2, 1.6)	1.2 (1.0, 1.4)	<0.001
LDL-C (mmol/L)	2.8 (2.4, 3.3)	2.6 (2.2, 3.0)	2.8 (2.4, 3.3)	3.0 (2.5, 3.4)	<0.001
AMH (ng/ml)	4.9 (2.5, 8.6)	6.0 (3.3, 9.8)	4.9 (2.5, 8.5)	4.0 (2.0, 7.3)	<0.001
AFC	27.0 (14.0, 33.0)	29.0 (17.0, 33.0)	26.0 (14.0, 33.0)	24.0 (13.0, 33.0)	<0.001
EM (mm)	12.2 (10.9, 13.6)	12.2 (10.9, 13.5)	12.1 (10.8, 13.5)	12.2 (10.8, 13.6)	0.003
Data in embryo transfer cycle					
Methods of ART					<0.001
IVF	31,430 (67.8)	10,489 (66.8)	10,240 (67.5)	10,701 (69.2)	
ICSI	7,458 (16.1)	2,721 (17.3)	2,394 (15.8)	2,343 (15.1)	
IVF+ICSI	3,382 (7.3)	1,172 (7.5)	1,095 (7.2)	1,115 (7.2)	
PGT-A	4,067 (8.8)	1,320 (8.4)	1,439 (9.5)	1,308 (8.5)	
Stimulation protocol					<0.001
Agonist	34,731 (75.0)	12,224 (77.8)	11,345 (74.8)	11,162 (72.2)	
Antagonist	7,280 (15.7)	2,163 (13.8)	2,441 (16.1)	2,676 (17.3)	
Others	4,326 (9.3)	1,315 (8.4)	1,382 (9.1)	1,629 (10.5)	
Donated sperm	2,573 (5.6)	944 (6.0)	827 (5.5)	802 (5.2)	0.005
Blastocyst transfer	14,695 (31.7)	5,539 (35.3)	4,917 (32.4)	4,239 (27.4)	<0.001

Tertile 1: ≤177; tertile 2: (177,203]; tertile 3: >203. All continuous variable showed a non-normal distribution after Anderson–Darling test. Data are expressed as median (interquartile range) for non-normally distributed continuous variables. Categorical variables were expressed in frequency (%). TyG-BMI, triglyceride glucose-body mass index; PCOS, polycystic ovary syndrome; BMI, body mass index; SBP, systolic blood pressure; DBP, diastolic blood pressure; TC, total cholesterol; HDL-C, high-density lipoprotein cholesterol; LDL-C, low-density lipoprotein cholesterol; AMH, anti-Müllerian hormone; AFC, antral follicle count; EM, endometrium; ART, assisted reproductive technology; IVF, *in vitro* fertilization; ICSI, intracytoplasmic sperm injection; PGT-A, preimplantation genetic testing for aneuploidy.

Baseline characteristics across TyG-BMI tertiles in the first transfer cycle analysis set are shown in **Supplementary Table 2**. Among 45,438 participants in the first transfer cycle analysis set (mean age 30.0 years), 15,624 (34.4%) were of low TyG-BMI, 14,783 (32.5%) were of medium TyG-BMI, and 15,031 (33.1%) were of high TyG-BMI. Baseline characteristics across TyG-BMI tertiles exhibited consistent trends with those in the cumulative live birth analysis set.

### Association of TyG-BMI with assisted pregnancy outcomes

Univariate and multivariable analyses between the tertiles of the TyG-BMI and assisted reproductive results are presented in **Tables 2** and **3**, respectively. Overall, a higher TyG-BMI level showed lower incidences of CLBR and adverse reproductive outcomes. CLBRs in T1 vs. T2 vs. T3 groups were 64.9% vs. 59.6% vs. 56.2% (**Table 2**). In **Table 3**, compared with individuals in the T1 group, those in the T2 group (aRR, 0.990; 95%CI, 0.974 to 1.007; aAR, −0.006; 95%CI, −0.024 to 0.012) and T3 group (aRR, 0.973; 95%CI, 0.956 to 0.991; aAR, −0.015; 95%CI, −0.033 to 0.003) showed a lower risk of CLBR.

**Table 2:** Results on pregnancy according to different categories of cycle by tertiles of TyG-BMI.

Categories of cycle	Total	Tertile1 ≤177	Tertile2 (177, 203]	Tertile3 >203	P
Cumulative live birth cycle, n	46337	15702	15168	15467	
Cumulative live birth	27,923 (60.3)	10,190 (64.9)	9,046 (59.6)	8,687 (56.2)	<0.001
First transplant cycle, n	45438	15624	14783	15031	
Type of embryo transfer cycle					<0.001
Fresh	28,648 (63.0)	9,572 (61.3)	9,411 (63.7)	9,665 (64.3)	
Frozen	16,790 (37.0)	6,052 (38.7)	5,372 (36.3)	5,366 (35.7)	
Clinical pregnancy	30,565 (67.0)	10,631 (68.0)	10,246 (66.7)	9,688 (66.3)	0.005
Live birth	26,053 (57.1)	9,295 (59.5)	8,749 (57.0)	8,009 (54.8)	<0.001
Clinical pregnancy cycle, n	30539	10629	9896	10014	
First trimester miscarriage	3,109 (10.2)	969 (9.1)	1,004 (10.1)	1,136 (11.3)	<0.001
Second- or third-trimester fetal loss	1,356 (4.4)	356 (3.3)	426 (4.3)	574 (5.7)	<0.001
Live birth cycle, n	26043	9296	8458	8289	
Delivery method					<0.001
Natural birth	6,512 (25.0)	2,840 (30.6)	2,042 (24.1)	1,630 (19.7)	
Cesarean section	19,531 (75.0)	6,456 (69.4)	6,416 (75.9)	6,659 (80.3)	
Gestational age (weeks)	38.6 (37.3, 39.6)	38.7 (37.3, 39.6)	38.6 (37.3, 39.6)	38.4 (37.1, 39.4)	<0.001
Number of newborns					0.616
1	19,050 (73.1)	6,833 (73.5)	6,165 (72.9)	6,052 (73.0)	
≥2	6,993 (26.9)	2,463 (26.5)	2,293 (27.1)	2,237 (27.0)	
Gestational diabetes	4,411 (16.9)	1,171 (12.6)	1,507 (17.8)	1,733 (20.9)	<0.001
Gestational hypertension	1,003 (3.9)	185 (2.0)	309 (3.7)	509 (6.1)	<0.001
Preterm birth	5,135 (19.7)	1,658 (17.8)	1,647 (19.5)	1,830 (22.1)	<0.001
Neonatal malformation	368 (1.4)	138 (1.5)	112 (1.3)	118 (1.4)	0.661

All continuous variable showed a non-normal distribution after Anderson–Darling test. Data are expressed as median (interquartile range) for non-normally distributed continuous variables. Categorical variables were expressed in frequency (%).

**Table 3:** Multivariate modified Poisson regression analysis for reproductive outcomes by tertiles of TyG-BMI.

Outcomes	TyG-BMI	aRR (95%CI)	aAR (95%CI)	aAR% (95%CI)
Cumulative live birth	T1	Ref	Ref	Ref
	T2	0.990 (0.974, 1.007)	−0.006 (−0.024, 0.012)	−1.055 (−4.057, 1.948)
	T3	0.973 (0.956, 0.991)	−0.015 (−0.033, 0.003)	−2.528 (−5.588, 0.531)
	P for trend	0.006	–	–
Clinical pregnancy	T1	Ref	Ref	Ref
	T2	1.017 (1.001, 1.033)	0.013 (−0.004, 0.030)	1.935 (−0.657, 4.526)
	T3	1.031 (1.015, 1.048)	0.023 (0.006, 0.040)	3.451 (0.939, 5.963)
	P for trend	<0.001	–	–
Live birth	T1	Ref	Ref	Ref
	T2	1.004 (0.985, 1.024)	0.003 (−0.015, 0.020)	0.455 (−2.644, 3.555)
	T3	0.991 (0.972, 1.011)	−0.004 (−0.022, 0.013)	−0.799 (−3.952, 2.354)
	P for trend	0.419	–	–
First-trimester miscarriage	T1	Ref	Ref	Ref
	T2	1.016 (0.934, 1.106)	0.001 (−0.010, 0.013)	1.584 (−11.366, 14.535)
	T3	1.090 (1.002, 1.185)	0.008 (−0.004, 0.020)	8.442 (−3.468, 20.352)
	P for trend	0.034	–	–
Second- or third-trimester fetal loss	T1	Ref	Ref	Ref
	T2	1.278 (1.112, 1.468)	0.008 (0.001, 0.015)	21.872 (5.628, 38.116)
	T3	1.694 (1.481, 1.938)	0.021 (0.013, 0.029)	41.233 (29.388, 53.078)
	P for trend	<0.001	–	–
Macrosomia	T1	Ref	Ref	Ref
	T2	1.578 (1.372, 1.814)	0.022 (0.012, 0.033)	36.986 (23.629, 50.342)
	T3	2.352 (2.060, 2.685)	0.052 (0.039, 0.065)	58.121 (49.615, 66.627)
	P for trend	<0.001	–	–
Preterm birth	T1	Ref	Ref	Ref
	T2	1.107 (1.041, 1.178)	0.020 (0.002, 0.038)	9.687 (1.332, 18.042)
	T3	1.244 (1.170, 1.323)	0.045 (0.026, 0.064)	19.559 (12.284, 26.833)
	P for trend	<0.001	–	–

RRs, risk ratios; ARs, absolute risk; T, tertiles (T1: ≤177; T2: (177,203]; T3: >203); CI, confidence interval; Ref, reference; TyG-BMI, triglyceride glucose-body mass index. The model was adjusted for female age, education level, smoking, alcoholism, hypothyroidism, polycystic ovary syndrome, anti-Müllerian hormone, pre-pregnancy hypertension, pre-pregnancy diabetes, gravidity, and parity.

In addition, a higher TyG-BMI was associated with a modest but statistically significant increase in the probability of reaching embryo transfer (**Supplementary Table 3**). Compared with the lowest tertile, the adjusted risk ratios were 1.012 (95% CI, 1.004–1.021) for both T2 and T3.

Analyses on secondary outcomes found that the T3 group exhibited higher incidences in clinical pregnancy (aRR, 1.026; 95%CI, 1.010 to 1.044), first-trimester miscarriage (aRR, 1.119; 95%CI, 1.026 to 1.220), second- or third-trimester fetal loss (aRR, 1.634; 95%CI, 1.423 to 1.876), macrosomia (aRR, 2.405; 95%CI, 2.063 to 2.803), and preterm birth (aRR, 1.157; 95%CI, 1.084 to 1.236), when compared with that of the T1 group. The absolute risk increases were modest for most outcomes (approximately 1–2 percentage points), whereas larger increases were observed for macrosomia and preterm birth, with absolute risk differences of approximately 5.2 percentage points (aAR, 0.052; 95% CI, 0.039 to 0.065) and 4.5 percentage points (aAR, 0.045; 95% CI, 0.026 to 0.064), respectively. No significant associations were observed between the tertiles of the TyG-BMI and live birth, number of newborns, and neonatal malformation (*P* > 0.05).

### The dose–response relationships between TyG-BMI and assisted pregnancy outcomes

CLBR was negatively associated with the TyG-BMI (*P*-overall = 0.029, *P*-non-linear = 0.144) (**Figure 2B**). An SD increase in TyG-BMI was associated with a 0.9% (aRR per SD, 0.991; 95%CI, 0.984-0.999) reduction in CLBR (**Figure 2B**).

There was a linear positive association observed between the TyG-BMI and clinical pregnancy (*P*-overall < 0.001, *P*-non-linear =0.444) (**Figure 2C**) and second- or third-trimester fetal loss (*P*-overall < 0.001, *P*-non-linear = 0.052) (**Figure 2F**). Additionally, positive non-linear association was observed between the TyG-BMI and preterm birth (*P*-overall < 0.001, *P*-non-linear = 0.005) (**Figure 2G**) and macrosomia (*P*-overall < 0.001, *P*-non-linear = 0.005) (**Figure 2H**).

### Subgroup analyses

**Figure 3:**
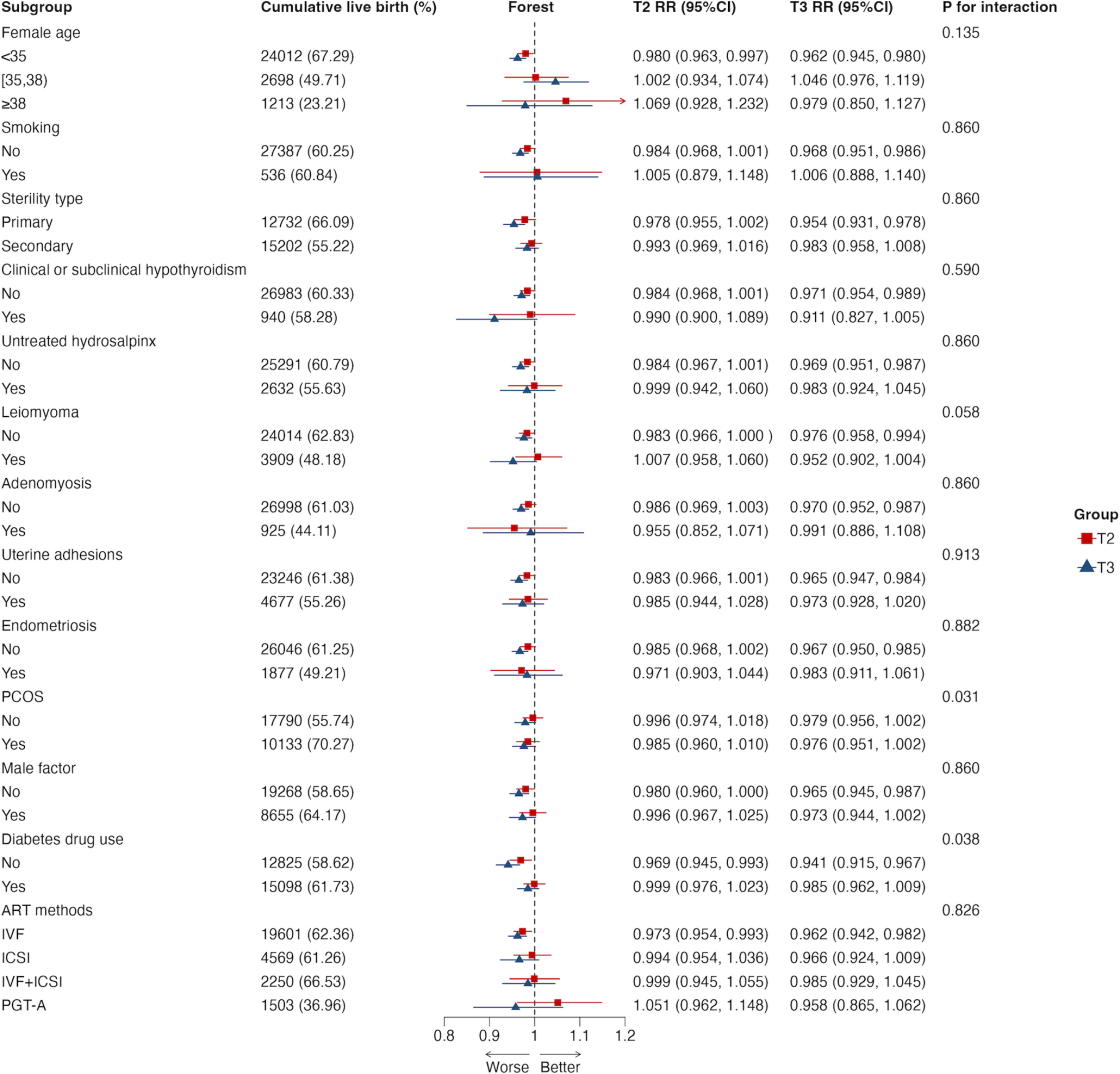
Forest plot of subgroup analysis on the association of triglyceride glucose-body mass index and cumulative live birth rate. Adjustment factors included female age, education level, smoking, alcoholism, hypothyroidism, polycystic ovary syndrome, anti-Müllerian hormone, pre-pregnancy hypertension, pre-pregnancy diabetes, gravidity, and parity. Group T1 served as the reference group.

Results from subgroup analyses on TyG-BMI and CLBR showed statistically significant differences between the PCOS subgroup and diabetes drug use subgroup (**Figure 3**). The protective association demonstrated a notably heightened magnitude among individuals with PCOS (*P* interaction = 0.031) and individuals who did not use diabetes drug (*P* interaction = 0.038).

Subgroup analyses for secondary outcomes are shown in **Supplementary Figures 3–8**. Positive associations between the TyG-BMI and clinical pregnancy were significantly more pronounced among individuals with frozen embryo transfer (*P* interaction = 0.031) and individuals underwent IVF+ICSI (*P* interaction = 0.025). A negative association between the TyG-BMI and live birth was more pronounced among individuals older than 38 years (*P* interaction = 0.044). There was no evidence of effect modification across subgroups in first-trimester miscarriage, second- or third-trimester fetal loss, macrosomia, and preterm birth (all P interaction > 0.05).

### Sensitivity analyses

Sensitivity analyses restricting the exposure assessment window to ≤3 and ≤6 months before oocyte retrieval yielded results consistent with the primary analyses across all outcomes (**Supplementary Tables 4**, **5**). For CLBR, the adjusted risk ratios for the highest TyG-BMI tertile (T3) were 0.969 (95% CI, 0.946–0.993) for the ≤3-month window and 0.981 (95% CI, 0.963–1.000) for the ≤6-month window; the direction and magnitude of the associations were comparable with the primary analysis, supporting the robustness of the findings.

## Discussion

This large-scale cohort study investigated the associations between the TyG-BMI and assisted pregnancy outcomes in women undergoing ART treatment. Our results revealed that a higher TyG-BMI was significantly associated with a lower CLBR. Furthermore, we identified positive dose–response relationships between the TyG-BMI and the incidence of reproductive outcomes following the first transfer cycle, including clinical pregnancy, second- or third-trimester fetal loss, and macrosomia and preterm birth. However, no significant association was observed between the TyG-BMI and live birth. These findings suggest that the TyG-BMI may be a potential marker associated with adverse outcomes. Importantly, when interpreted in terms of absolute effect measures, relatively larger absolute increases were observed for outcomes such as macrosomia and preterm birth, indicating that these may be of greater clinical relevance. To our knowledge, this is the first large-scale study to explore the potential predictive utility of TyG-BMI for ART success in a general infertility population.

In recent years, while the prognostic value of TyG-BMI in cardiovascular disease is gaining acceptance (26–28), its role in reproductive health remains less explored. Existing studies on TyG-BMI and pregnancy outcomes are limited. In the general obstetric population, Li et al. (29) demonstrated an association between elevated TyG-BMI and large-for-gestational-age infants, aligning with our finding of a positive association between high TyG-BMI and macrosomia. Given the clinical overlap between macrosomia and large-for-gestational-age infants (30), this suggests that TyG-BMI may influence fetal overgrowth through shared mechanisms (such as insulin resistance). Similarly, a large retrospective cohort study involving 67,936 pregnant women in China showed that women of pre-pregnancy overweight/obesity with a high TyG index were at a higher risk for preterm birth (11), which is also consistent with our results. Nonetheless, we provided additional evidence on population underwent ART.

To date, only two studies have examined the impact of the TyG-BMI in ART populations, both of which were restricted to patients with polycystic ovary syndrome (20, 21) and reported conflicting results. Our results in the polycystic ovary syndrome subgroup aligned with those of Li et al. (21), who similarly reported no significant differences in live birth rates across TyG-BMI quartiles. In contrast, Wu et al. (20) observed a stable inverse association between TyG-BMI and live birth. These discrepancies may be partly attributable to differences in ovarian stimulation protocols (31–33) and embryo transfer strategies (34–36).

In addition, a recent study by Song et al. (22) reported that higher TyG-BMI levels were associated with a higher risk of miscarriage and a lower CLBR, findings that are directionally consistent with our results. However, the magnitude of the associations reported by Song et al. was notably larger than those observed in our analysis. One plausible explanation for this discrepancy lies in the choice of association measure. Specifically, Song et al. relied on odds ratios derived from logistic regression, whereas we estimated risk ratios. When the outcome of interest is common in the study population (>10%), odds ratios tend to overestimate the corresponding risk ratios and therefore may exaggerate the strength of associations (37).

We observed a weak positive association between TyG-BMI and early reproductive outcomes (reaching embryo transfer and clinical pregnancy), which may be attributed to the metabolic environment associated with insulin resistance, especially the role of hyperleptinemia (38, 39). Existing research has indicated that leptin promoted cytotrophoblast invasion in a positive dose–response manner during early pregnancy, thereby facilitating embryo implantation (40). However, the invasive capacity of cytotrophoblasts gradually declines with exposure to higher levels of leptin in the second trimester of pregnancy and could create a situation where the trophoblasts fail to invade to a physiologic depth (40). Based on this evidence, we hypothesize that the metabolic state related to high TyG-BMI may transiently facilitate embryo implantation and early pregnancy establishment. Nevertheless, the persistence of such an aberrant metabolic environment proves detrimental to embryonic development and placental function, ultimately leading to a higher miscarriage rate and reduced live birth rate, which is consistent with our findings.

Another important observation from this study is the overall consistency of associations across multiple reproductive outcomes, suggesting that TyG-BMI may capture a common underlying metabolic disturbance influencing different stages of the assisted reproduction process. As a surrogate marker of insulin resistance, TyG-BMI may exert systemic effects on reproductive physiology through several interconnected biological pathways. At the ovarian level, insulin resistance may impair oocyte quality and developmental competence by increasing oxidative stress and inducing mitochondrial dysfunction (41, 42). At the endometrial level, insulin signaling plays a critical role in regulating cellular proliferation, differentiation (43, 44), and glucose utilization (45). Insulin resistance is a defect in signal transduction, which may reduce endometrial glucose uptake (46), alter decidualization processes (47, 48), and impair embryo–uterine crosstalk (49), thereby compromising endometrial receptivity (50). Additionally, insulin resistance consistently induces endothelial dysfunction through impaired signaling pathways that reduce nitric oxide bioavailability (51, 52), as well as induce abnormal angiogenic signals (53, 54), thereby directly compromises early placental vascularization. These mechanisms together provide a plausible biological basis linking elevated TyG-BMI with reduced likelihood of successful live birth and higher risk of adverse pregnancy outcomes such as miscarriage and preterm birth. Overall, these findings suggest that TyG-BMI may influence distinct stages of the assisted reproductive process through shared interconnected biological pathways. However, the clinical relevance of these findings remains to be established in future studies.

Given the consistent associations across outcomes, it is important to understand why TyG-BMI is particularly capable of capturing this underlying metabolic disturbance. From a biological perspective, insulin resistance is characterized by the interplay between dysregulated glucose–lipid metabolism and adipose tissue expansion and dysfunction (55, 56). The TyG index primarily reflects peripheral insulin resistance based on fasting triglycerides and glucose, whereas BMI captures obesity-related components of insulin resistance, such as excess adiposity (57), free fatty acid flux (58), and adipokine imbalance (59). By integrating these two dimensions, TyG-BMI may more comprehensively reflect the metabolic phenotype often described as “metabolically unhealthy obesity”. The use of a composite indicator incorporating both metabolic and anthropometric information may help to reduce potential misclassification arising from individuals with discordant metabolic and obesity status. From a practical perspective, BMI is routinely available without additional cost or testing burden. Combining BMI with TyG does not require extra measurements but may provide a more integrated characterization of metabolic status. Accordingly, TyG-BMI may offer a more comprehensive representation of insulin resistance-related metabolic disturbances, which is supported by existing evidence (16, 17).

The subgroup analyses indicated that the TyG-BMI holds greater significance for some specific subgroups of women with respect to the success rate of ART treatment. We identified that a negative association between the TyG-BMI and CLBR was significantly enhanced in individuals with PCOS in comparison with those that without PCOS, with a significant interaction between TyG-BMI and PCOS status, which may be explained by the intrinsic metabolic characteristics of this population. PCOS is characterized by underlying insulin resistance and metabolic dysfunction (60), which may amplify the detrimental effects of elevated TyG-BMI on reproductive processes, leading to a compounded negative impact on CLBR. Additionally, we also observed that a negative association between the TyG-BMI and CLBR was pronounced in individuals who did not use antidiabetic drugs, with a significant interaction between TyG-BMI and use of antidiabetic medications. Using hypoglycemic drugs appeared to alleviate the negative impact of high TyG-BMI on the CLBR. Nonetheless, all subgroup analyses should be interpreted as exploratory and warrant further confirmation in future studies.

This study has several strengths. Significantly, this is the first investigation of the TyG-BMI and assisted pregnancy outcomes in the general ART population. The substantial sample size allowed us to explore interactions between the TyG-BMI and several subgroups of participants with different characteristics. Additionally, there remains limited clinical evidence regarding the association between the TyG-BMI and CLBR, which is regarded as the primary indicator for evaluating the success of IVF (61). Our study further investigated the associations between TyG-BMI and CLBR, as well as other adverse assisted pregnancy outcomes, thereby providing additional evidence to support the potential role of TyG-BMI as a metabolic marker for the association with ART outcomes. Furthermore, we used DAGs to identify a minimally sufficient set of confounders, thereby strengthening the causal interpretability of our findings and reducing the risk of inappropriate covariate adjustment.

Nevertheless, it is important to take into account certain limitations. First, given the retrospective observational design of this study, causal inferences cannot be established, and the findings should be interpreted as associations rather than evidence of causality. Second, although this study adjusted for numerous potential confounders, and several alternative analytic approaches yielded similar results, potential residual bias due to unmeasured confounding (such as baseline lifestyle factors, including eating patterns and physical activity levels) cannot be entirely ruled out. Third, we observed that TyG-BMI was modestly associated with the probability of reaching embryo transfer, suggesting that analyses restricted to women who underwent embryo transfer may be subject to selection bias. Therefore, the associations observed for post-transfer outcomes should be interpreted as conditional on having undergone embryo transfer rather than as estimates of the total effect in the overall population. Fourth, the number of embryos transferred and embryo quality, both important determinants of ART outcomes, were not explicitly accounted for in the present analysis. These factors may act as potential mediators linking metabolic status to reproductive outcomes; however, mediation pathways were not formally examined in this study. Additionally, measurements of FPG, TG, and other parameters were only taken at baseline and could have potentially fluctuated throughout the follow-up period as a result of weight modifying interventions, lifestyle modifications and insulin-sensitizing medications. However, detailed longitudinal follow-up data were not available, which may limit our ability to capture dynamic trajectories over time and to fully assess their potential impact on the observed association. Future studies are suggested to incorporate more information across multiple time points to better account for time-varying confounding and the impact of trajectory changes on the associations. Finally, the external generalizability of our findings should be interpreted with caution. Compared with the data of Western populations undergoing fertility treatment in previous literature (62), women in our Chinese cohort had a lower proportion of overweight and obesity but a higher prevalence of metabolic-related conditions such as polycystic ovary syndrome. This pattern is consistent with previous evidence suggesting that Asian populations tend to have a lower BMI but greater susceptibility to metabolic abnormalities compared with European populations (63, 64). Given that data from Western populations were not available in the present study, caution is warranted when extrapolating our findings to diverse international populations.

Our study represents a preliminary exploration of the associations between maternal baseline TyG-BMI and ART outcomes. Future research should further investigate the potential mechanisms underlying these associations, particularly by formally examining the mediating roles of key reproductive factors such as the number of embryos transferred and embryo quality. In addition, exploring the TyG-BMI at different time points (e.g., early pregnancy or later stages of pregnancy) as well as their dynamic changes over time may provide more nuanced insights into the potential associations with ART outcomes. Furthermore, multicenter studies with large sample sizes are needed to better establish causality between TyG-BMI and ART outcomes. Such efforts would generate more robust evidence to better guide clinical practice in the field of assisted reproduction.

## Conclusion

In this large-scale study, our study revealed that a higher TyG-BMI is associated with a lower CLBR, as well as a higher risk of miscarriage, preterm birth, and macrosomia, which suggests that the TyG-BMI may be a potential indicator for adverse assisted reproductive outcomes. Prospective studies are warranted to confirm these associations and explore the utility of TyG-BMI in clinical risk stratification and management strategies for ART patients.

## Data Availability

The raw data supporting the conclusions of this article will be made available by the authors, without undue reservation.
