# Supplementary material for: Elevated triglyceride glucose-body mass index is associated with a higher risk of reduced cumulative live birth and adverse pregnancy outcomes in women undergoing assisted reproductive technology: a retrospective cohort study
Source: Front Endocrinol (Lausanne). 2026 Jun 17;17:1842023. doi: 10.3389/fendo.2026.1842023 (PMC13318790; doi:10.3389/fendo.2026.1842023)
Supplement: Supplementary file 1 [file Presentation1.pdf]

Table S1. Numbers and percentages of missing variables at baseline.

Table S2. Baseline characteristics of participants by tertiles of TyG-BMI index in the first transfer cycle analysis set.

Table S3. Multivariate modified Poisson regression analysis for reaching embryo transfer by tertiles of TyG-BMI.

Table S4. Sensitivity analysis of interval time between BMI/FPG/TG measurements and oocyte retrieval ⩽ 3 months.

Table S5. Sensitivity analysis of interval time between BMI/FPG/TG measurements and oocyte retrieval ⩽ 6 months.

Figure S1. Directed acyclic graph (DAG) for the assumed causal relationships between TyG-BMI index and Pregnancy outcomes (Cumulative live birth, Clinical pregnancy, Live birth and miscarriage).

Figure S2. Directed acyclic graph (DAG) for the assumed causal relationships between TyG-BMI index and Neonatal outcomes (Preterm birth and Macrosomia).

Figure S3. Subgroup group analysis for Clinical Pregnancy in the first transfer cycle of participants by tertiles of TyG-BMI.

Figure S4. Subgroup group analysis for Live Birth in the first transfer cycle of participants by tertiles of TyG-BMI.

Figure S5. Subgroup group analysis for First trimester miscarriage in the first transfer cycle of participants by tertiles of TyG-BMI.

Figure S6. Subgroup group analysis for 2nd or 3rd trimester fetal loss in the first transfer cycle of participants by tertiles of TyG-BMI.

Figure S7. Subgroup group analysis for Macrosomia in the first transfer cycle of participants by tertiles of TyG-BMI.

Figure S8. Subgroup group analysis for Preterm Birth in the first transfer cycle of participants by tertiles of TyG-BMI.

Tables

Table S1. Numbers and percentages of missing variables at baseline.

Variables	Number	Percentage (%)
Female age	0	0
Male age	43	0.080
BMI	0	0
Age of menarche	72	0.134
Education	68	0.126
Current smoking	13	0.024
Alcoholism	13	0.024
Years of infertility	2	0.004
Sterility type	0	0
Fallopian tube abnormalities	0	0
Male factor	0	0
PCOS	0	0
Uterine adhesions	0	0
Adenomyosis	0	0
Leiomyoma	0	0
Endometriosis	0	0
Untreated hydrosalpinx	0	0
Chocolate cyst	0	0
Recurrent miscarriage	0	0
parity	15	0.028
gravidity	14	0.026
TyG-BMI	0	0
AFC	0	0
AMH	21	0.039
EM	153	0.284
LDL-C	0	0
HDL-C	0	0
TC	0	0
SBP	25	0.046
DBP	26	0.048
Methods of ART	0	0
Promoting plan	0	0
Donated sperm	0	0
Blastocyst transfer	0	0
Gestational age	100	0.186
Delivery method	54	0.100
Pre-pregnancy hypertension	0	0
Pre-pregnancy diabetes	0	0

Abbreviations: AFC, antral follicle count; AMH, antimullerian hormone; ART, assisted reproductive technology; BMI, body mass index; DBP, diastolic blood pressure; EM, endometrium; HDL-C, high-density lipoprotein cholesterol; LDL-C, low-density lipoprotein cholesterol; PCOS, polycystic ovary syndrome; SBP, systolic blood pressure; TC, total cholesterol; TyG-BMI, triglyceride glucose-body mass index.

Table S2. Baseline characteristics of participants by tertiles of TyG-BMI index in the first transfer cycle analysis set.

Characteristics	Total	Tertile1 ≤177	Tertile2 (177, 203]	Tertile3 >203	*p*
No. of cases	45438	15624	14783	15031	
Male age (years), Median (IQR)	32.6 (29.9, 36.3)	31.8 (29.3, 35.0)	32.8 (29.9, 36.6)	33.4 (30.5, 37.5)	<0.001
**Maternal Variables**					
Female age (years), Median (IQR)	30.0 (28.0, 34.0)	30.0 (27.0, 32.0)	31.0 (28.0, 34.0)	31.0 (28.0, 35.0)	<0.001
< 35	36,609 (80.6)	13,724 (87.8)	11,691 (79.1)	11,194 (74.5)	<0.001
[35,38)	5,168 (11.4)	1,308 (8.4)	1,799 (12.2)	2,061 (13.7)	
≥ 38	3,661 (8.0)	592 (3.8)	1,293 (8.7)	1,776 (11.8)	
BMI (kg/m^2), Median (IQR)	22.5 (20.5, 24.3)	19.8 (18.8, 20.8)	22.6 (21.8, 23.4)	25.1 (24.0, 26.4)	<0.001
< 18.5	3,138 (6.9)	3,137 (20.1)	1 (0.0)	0 (0.0)	<0.001
18.5-24	28,881 (63.6)	12,483 (79.9)	12,930 (87.5)	3,468 (23.1)	
≥ 24	13,419 (29.5)	4 (0.0)	1,852 (12.5)	11,563 (76.9)	
Age at menarche (years), Median (IQR)	13.0 (13.0, 14.0)	13.0 (13.0, 14.0)	13.0 (13.0, 14.0)	13.0 (13.0, 14.0)	<0.001
Education, n (%)					<0.001
Primary school or below	21,744 (47.9)	6,310 (40.4)	7,086 (47.9)	8,348 (55.5)	
High school	6,888 (15.2)	2,457 (15.7)	2,261 (15.3)	2,170 (14.4)	
University level or above	16,806 (37.0)	6,857 (43.9)	5,436 (36.8)	4,513 (30.0)	
Infertility years, Median (IQR)	3.0 (1.0, 5.0)	3.0 (1.0, 4.0)	3.0 (1.0, 5.0)	3.0 (2.0, 5.0)	<0.001
Infertility type, n (%)					<0.001
Primary	19,375 (42.6)	7,104 (45.5)	6,022 (40.7)	6,249 (41.6)	
Secondary	26,063 (57.4)	8,520 (54.5)	8,761 (59.3)	8,782 (58.4)	
Female current smoking, n (%)					<0.001
No	44,473 (97.9)	15,365 (98.3)	14,500 (98.1)	14,608 (97.2)	
Yes	965 (2.1)	259 (1.7)	283 (1.9)	423 (2.8)	
Female alcoholism, n (%)					0.889
No	45,369 (99.8)	15,602 (99.9)	14,759 (99.8)	15,008 (99.8)	
Yes	69 (0.2)	22 (0.1)	24 (0.2)	23 (0.2)	
Hypertension, n (%)					<0.001
No	44,835 (98.7)	15,567 (99.6)	14,622 (98.9)	14,646 (97.4)	
Yes	603 (1.3)	57 (0.4)	161 (1.1)	385 (2.6)	
Diabetes, n(%)					<0.001
No	45,289 (99.7)	15,612 (99.9)	14,750 (99.8)	14,927 (99.3)	
Yes	149 (0.3)	12 (0.1)	33 (0.2)	104 (0.7)	
Clinical and subclinical hypothyroidism, n (%)					<0.001
No	43,858 (96.5)	15,187 (97.2)	14,283 (96.6)	14,388 (95.7)	
Yes	1,580 (3.5)	437 (2.8)	500 (3.4)	643 (4.3)	
Parity, n (%)					<0.001
0	35,857 (78.9)	13,122 (84.0)	11,500 (77.8)	11,235 (74.7)	
1	8,159 (18.0)	2,221 (14.2)	2,812 (19.0)	3,126 (20.8)	
⩾2	1,422 (3.1)	281 (1.8)	471 (3.2)	670 (4.5)	

Gravidity, n (%)					<0.001
0	19,254 (42.4)	7,062 (45.2)	5,985 (40.5)	6,207 (41.3)	
1	11,285 (24.8)	3,890 (24.9)	3,634 (24.6)	3,761 (25.0)	
⩾2	14,899 (32.8)	4,672 (29.9)	5,164 (34.9)	5,063 (33.7)	
Infertility related diseases					
PCOS, n (%)					<0.001
No	30,223 (66.5)	10,529 (67.4)	9,936 (67.2)	9,758 (64.9)	
Yes	15,215 (33.5)	5,095 (32.6)	4,847 (32.8)	5,273 (35.1)	
Male factor, n (%)					0.001
No	31,666 (69.7)	10,927 (69.9)	10,427 (70.5)	10,312 (68.6)	
Yes	13,772 (30.3)	4,697 (30.1)	4,356 (29.5)	4,719 (31.4)	
Uterine adhesions, n (%)					<0.001
No	37,370 (82.2)	12,631 (80.8)	12,081 (81.7)	12,658 (84.2)	
Yes	8,068 (17.8)	2,993 (19.2)	2,702 (18.3)	2,373 (15.8)	
Adenomyosis, n (%)					<0.001
No	42,631 (93.8)	14,866 (95.1)	13,877 (93.9)	13,888 (92.4)	
Yes	2,807 (6.2)	758 (4.9)	906 (6.1)	1,143 (7.6)	
Leiomyoma, n (%)					<0.001
No	37,795 (83.2)	13,493 (86.4)	12,180 (82.4)	12,122 (80.6)	
Yes	7,643 (16.8)	2,131 (13.6)	2,603 (17.6)	2,909 (19.4)	
Endometriosis, n (%)					0.063
No	40,946 (90.1)	14,127 (90.4)	13,343 (90.3)	13,476 (89.7)	
Yes	4,492 (9.9)	1,497 (9.6)	1,440 (9.7)	1,555 (10.3)	
Untreated hydrosalpinx, n (%)					<0.001
No	40,891 (90.0)	14,177 (90.7)	13,309 (90.0)	13,405 (89.2)	
Yes	4,547 (10.0)	1,447 (9.3)	1,474 (10.0)	1,626 (10.8)	
Chocolate cyst, n (%)					<0.001
No	44,447 (97.8)	15,189 (97.2)	14,473 (97.9)	14,785 (98.4)	
Yes	991 (2.2)	435 (2.8)	310 (2.1)	246 (1.6)	
Recurrent miscarriage, n (%)					<0.001
No	42,908 (94.4)	14,600 (93.4)	13,897 (94.0)	14,411 (95.9)	
Yes	2,530 (5.6)	1,024 (6.6)	886 (6.0)	620 (4.1)	
**Clinical data at baseline**					
TyG-BMI, Median (IQR)	189.3 (168.8, 210.3)	161.5 (151.6, 169.5)	189.8 (183.6, 196.3)	219.8 (210.5, 234.0)	<0.001
SBP (mmHg), Median (IQR)	115.0 (107.0, 122.0)	112.0 (105.0, 118.0)	115.0 (107.0, 121.0)	117.0 (110.0, 125.0)	<0.001
DBP (mmHg), Median (IQR)	75.0 (69.0, 80.0)	72.0 (67.0, 78.0)	75.0 (69.0, 80.0)	77.0 (70.0, 83.0)	<0.001
TC (mmol/L), Median (IQR)	4.3 (3.9, 4.8)	4.2 (3.8, 4.7)	4.3 (3.9, 4.8)	4.5 (4.0, 5.0)	<0.001
HDL-C (mmol/L), Median (IQR)	1.4 (1.2, 1.6)	1.6 (1.4, 1.8)	1.4 (1.2, 1.6)	1.2 (1.0, 1.4)	<0.001
LDL-C (mmol/L), Median (IQR)	2.8 (2.4, 3.2)	2.6 (2.2, 3.0)	2.8 (2.4, 3.3)	3.0 (2.5, 3.4)	<0.001
AMH (ng/ml), Median (IQR)	5.2 (2.9, 8.7)	6.1 (3.5, 9.8)	5.1 (2.9, 8.6)	4.3 (2.4, 7.5)	<0.001
AFC, Median (IQR)	28.0 (15.0, 35.0)	29.0 (17.0, 33.0)	27.0 (15.0, 35.0)	26.0 (14.0, 35.0)	<0.001
EM (mm), Median (IQR)	12.2 (10.9, 13.6)	12.2 (11.0, 13.5)	12.1 (10.9, 13.5)	12.3 (10.9, 13.6)	0.008
**Data in embryo transfer cycle**					
Methods of ART, n (%)					<0.001
IVF	31,571 (69.5)	10,619 (68.0)	10,231 (69.2)	10,721 (71.3)	

ICSI	7,387 (16.3)	2,713 (17.4)	2,382 (16.1)	2,292 (15.2)	
IVF+ICSI	3,643 (8.0)	1,267 (8.1)	1,168 (7.9)	1,208 (8.0)	
PGT-A	2,837 (6.2)	1,025 (6.6)	1,002 (6.8)	810 (5.4)	
Stimulation protocol, n (%)					<0.001
Agonist	35,767 (78.7)	12,523 (80.2)	11,650 (78.8)	11,594 (77.1)	
Antagonist	6,101 (13.4)	1,971 (12.6)	2,000 (13.5)	2,130 (14.2)	
Others	3,570 (7.9)	1,130 (7.2)	1,133 (7.7)	1,307 (8.7%)	
Donated sperm, n (%)					0.002
No	42,823 (94.2)	14,647 (93.7)	13,949 (94.4)	14,227 (94.7)	
Yes	2,615 (5.8)	977 (6.3)	834 (5.6)	804 (5.3)	
Blastocyst transfer, n (%)					<0.001
No	27,874 (61.3)	9,129 (58.4)	8,969 (60.7)	9,776 (65.0)	
Yes	17,564 (38.7)	6,495 (41.6)	5,814 (39.3)	5,255 (35.0)	

Note: All continuous variable showed a non-normal distribution after Anderson-Darling test. Data are expressed as median (interquartile range) for non-normally distributed continuous variables. Categorical variables were expressed in frequency or as a percentage.

Abbreviations: TyG-BMI = triglyceride glucose-body mass index; PCOS = polycystic ovary syndrome; BMI = body mass index; SBP = systolic blood pressure; DBP = diastolic blood pressure; TC = total cholesterol; HDL-C = high-density lipoprotein cholesterol; LDL-C = low-density lipoprotein cholesterol; AMH = antimullerian hormone; AFC = antral follicle count; EM = endometrium; ART = assisted reproductive technology; IVF = In Vitro Fertilization; ICSI = Intracytoplasmic Sperm Injection; PGT-A = Preimplantation Genetic Testing for Aneuploidy.

Table S3. Multivariate modified Poisson regression analysis for reaching embryo transfer by tertiles of TyG-BMI.

Outcomes	TyG-BMI	aRR (95%CI)	aAR (95%CI)	aAR% (95%CI)
Reaching	T1	Ref	Ref	Ref
embryo	T2	**1.012 (1.004, 1.021)**	**0.011 (0.001, 0.022)**	**1.267 (0.135, 2.400)**
transfer	T3	**1.012 (1.004, 1.021)**	**0.013 (0.003, 0.023)**	**1.430 (0.311, 2.548)**

Note: RRs, risk ratios. T, tertiles (T1: ≤177; T2: (177,203]; T3: >203); CI, confidence interval; Ref, reference; TyG-BMI, triglyceride glucose-body mass index. Model was adjusted for female age, male age, education level, smoking, alcoholism, hypothyroidism, polycystic ovary syndrome, antimullerian hormone, pre-pregnancy hypertension, pre-pregnancy diabetes, gravidity and parity.

Table S4. Sensitivity analysis of interval time between BMI/FPG/TG measurements and oocyte retrieval ≤ 3 months.

Outcomes	T1	T2 aRR (95%CI)	T3 aRR (95%CI)
Cumulative Live Birth	Ref	0.993 (0.972, 1.016)	**0.969 (0.946, 0.993)**
Clinical Pregnancy	Ref	**1.023 (1.002, 1.043)**	**1.037 (1.015, 1.059)**
Live Birth	Ref	1.012 (0.987, 1.036)	0.996 (0.970 , 1.022)
First trimester miscarriage	Ref	0.997 (0.895, 1.110)	1.114 (0.999, 1.242)
2nd or 3rd trimester fetal loss	Ref	**1.328 (1.104, 1.598)**	**1.706 (1.422, 2.046)**
Preterm Birth	Ref	**1.149 (1.061, 1.245)**	**1.261 (1.162, 1.368)**
Macrosomia	Ref	**1.428 (1.199, 1.702)**	**2.225 (1.884, 2.628)**

Note: RRs, risk ratios. T, tertiles (T1: ≤177; T2: (177,203]; T3: >203); CI, confidence interval; Ref, reference; TyG-BMI, triglyceride glucose-body mass index. the adjusted factors included female age, education level, smoking, alcoholism, hypothyroidism, polycystic ovary syndrome, antimullerian hormone, pre-pregnancy hypertension, pre-pregnancy diabetes, gravidity and parity.

Table S5. Sensitivity analysis of interval time between BMI/FPG/TG measurements and oocyte retrieval ≤ 6 months.

Outcomes	T1	T2 aRR (95%CI)	T3 aRR (95%CI)
Cumulative Live Birth	Ref	0.992 (0.974, 1.009)	**0.981 (0.963, 1.000)**
Clinical Pregnancy	Ref	**1.020 (1.003, 1.037)**	**1.038 (1.020, 1.055)**
Live Birth	Ref	1.006 (0.987, 1.026)	0.998 (0.977, 1.018)
First trimester miscarriage	Ref	1.021 (0.935, 1.114)	**1.097 (1.004, 1.198)**
2nd or 3rd trimester fetal loss	Ref	**1.307 (1.129, 1.513)**	**1.695 (1.470, 1.954)**
Preterm Birth	Ref	**1.097 (1.029, 1.170)**	**1.231 (1.154, 1.313)**
Macrosomia	Ref	**1.563 (1.352, 1.806)**	**2.345 (2.043, 2.691)**

Note: RRs, risk ratios. T, tertiles (T1: ≤177; T2: (177,203]; T3: >203); CI, confidence interval; Ref, reference; TyG-BMI, triglyceride glucose-body mass index. the adjusted factors included female age, education level, smoking, alcoholism, hypothyroidism, polycystic ovary syndrome, antimullerian hormone, pre-pregnancy hypertension, pre-pregnancy diabetes, gravidity and parity.

Figures

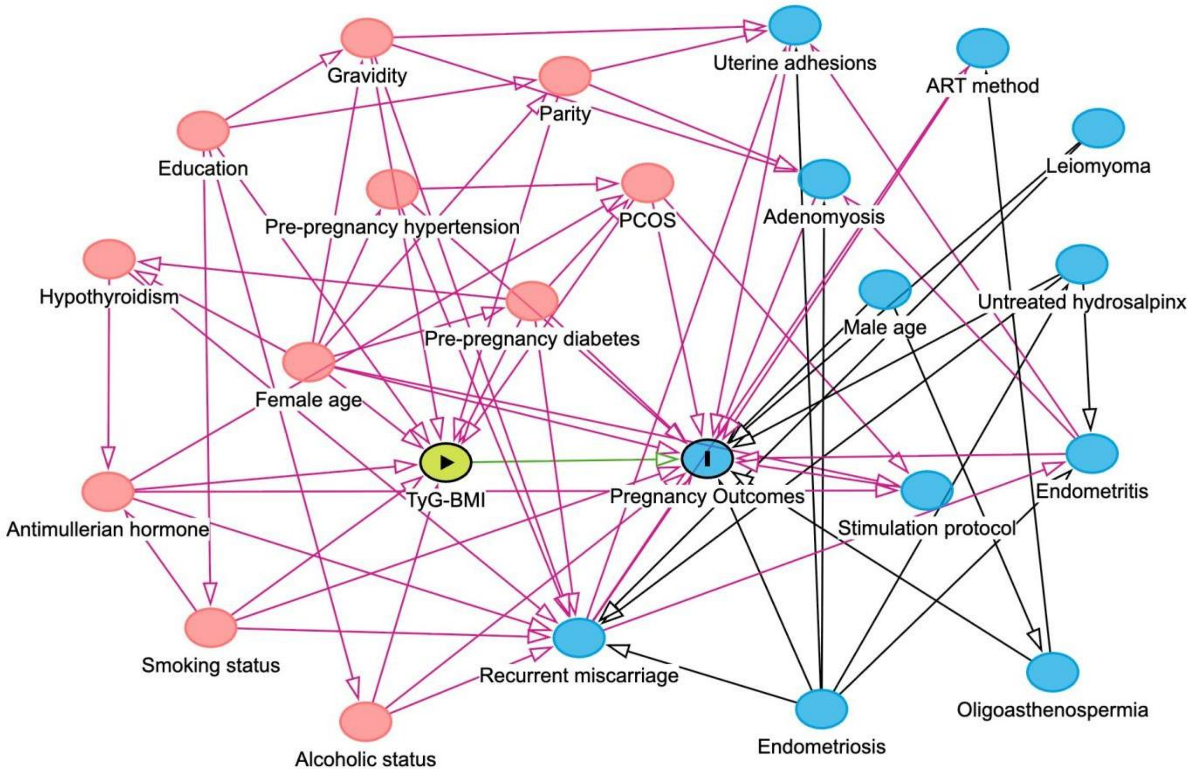

Figure S1. Directed acyclic graph (DAG) for the assumed causal relationships between TyG-BMI index and Pregnancy outcomes (Cumulative live birth, Clinical pregnancy, Live birth and miscarriage).

Note: PCOS = polycystic ovary syndrome; TyG-BMI = triglyceride glucose-body mass index.

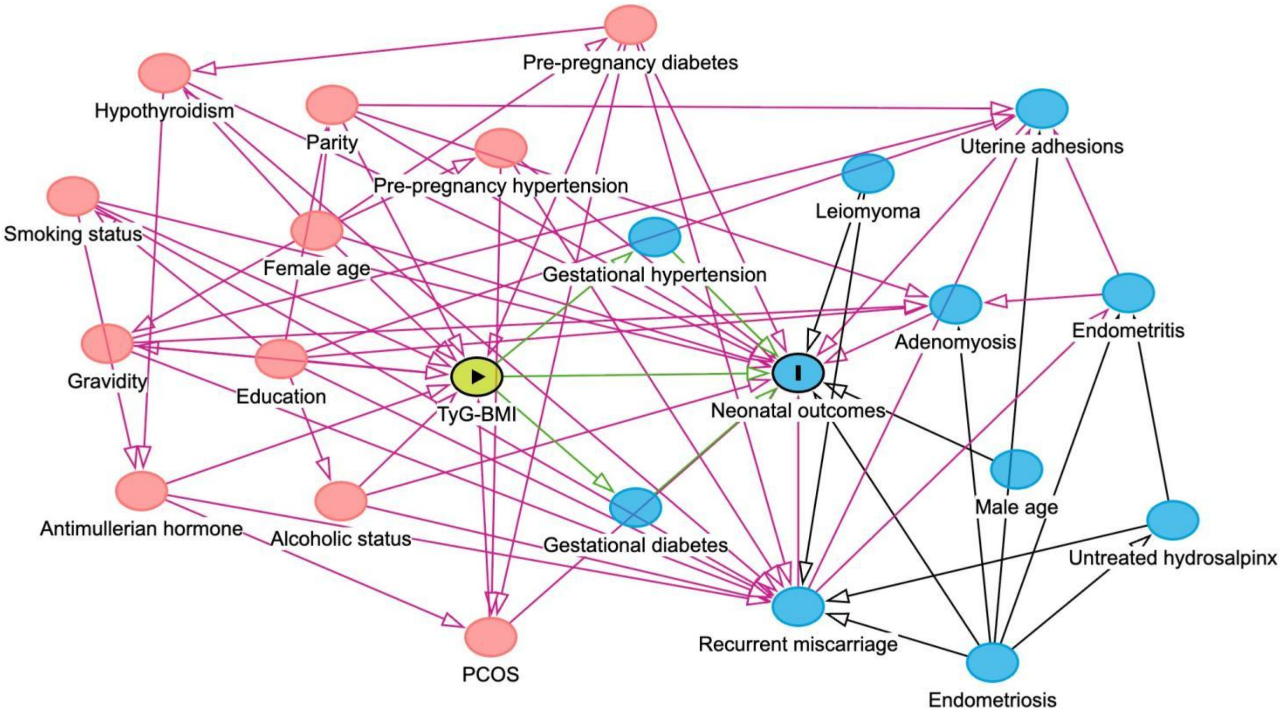

Figure S2. Directed acyclic graph (DAG) for the assumed causal relationships between TyG-BMI index and Neonatal outcomes (Preterm birth and Macrosomia).

Note: PCOS = polycystic ovary syndrome; TyG-BMI = triglyceride glucose-body mass index.

Subgroup	Clinical Pregnancy (%)	T2 RR (95%CI)	T3 RR (95%CI)	P for interaction
Female age				0.071
<35	25718 (70.25)	1.013 (0.997, 1.030)	1.023 (1.006, 1.040)	
[35,38)	3119 (60.35)	0.982 (0.926, 1.042)	1.066 (1.008, 1.129)	
≥38	1702 (46.49)	1.085 (0.978, 1.203)	1.040 (0.939, 1.152)	
Smoking				0.664
No	29888 (67.20)	1.012 (0.996, 1.028)	1.026 (1.010, 1.043)	
Yes	651 (67.46)	1.082 (0.960, 1.221)	1.119 (1.000, 1.252)	
Sterility type				0.664
Primary	13605 (70.22)	1.022 (0.999, 1.045)	1.021 (0.998, 1.045)	
Secondary	16934 (64.97)	1.007 (0.986, 1.029)	1.033 (1.010, 1.056)	
Clinical or subclinical hypothyroidism				0.664
No	29528 (67.33)	1.011 (0.995, 1.027)	1.027 (1.011, 1.044)	
Yes	1011 (63.99)	1.086 (0.990, 1.193)	1.054 (0.960, 1.157)	
Untreated hydrosalpinx				0.664
No	2796 (67.73)	1.015 (0.999, 1.032)	1.030 (1.014, 1.048)	
Yes	2843 (62.52)	0.993 (0.940, 1.049)	1.002 (0.948, 1.060)	
Leiomyoma				0.905
No	25706 (68.01)	1.014 (0.998, 1.032)	1.027 (1.010, 1.045)	
Yes	4833 (63.23)	1.010 (0.968, 1.054)	1.036 (0.993, 1.081)	
Adenomyosis				0.710
No	28762 (67.47)	1.014 (0.998, 1.030)	1.026 (1.010, 1.043)	
Yes	1777 (63.31)	0.998 (0.927, 1.074)	1.046 (0.975, 1.121)	
Uterine adhesions				0.334
No	25425 (68.04)	1.016 (0.999, 1.033)	1.022 (1.004, 1.040)	
Yes	5114 (63.39)	0.998 (0.959, 1.038)	1.048 (1.006, 1.091)	
Endometriosis				0.664
No	27686 (67.62)	1.011 (0.995, 1.028)	1.024 (1.007, 1.041)	
Yes	2853 (63.51)	1.032 (0.977, 1.090)	1.065 (1.008, 1.125)	
PCOS				0.772
No	19519 (64.58)	1.023 (1.003, 1.044)	1.040 (1.018, 1.062)	
Yes	11020 (72.43)	1.003 (0.979, 1.028)	1.017 (0.993, 1.042)	
Male factor				0.665
No	21192 (66.92)	1.006 (0.988, 1.025)	1.022 (1.002, 1.041)	
Yes	9347 (67.87)	1.030 (1.002, 1.060)	1.041 (1.012, 1.071)	
Type of embryo transfer cycle				0.031
Fresh	19692 (68.74)	1.010 (0.992, 1.030)	1.016 (0.997, 1.036)	
Frozen	10847 (64.60)	1.013 (0.986, 1.041)	1.046 (1.018, 1.075)	
Diabetes drug use				0.772
No	14129 (66.57)	1.018 (0.996, 1.041)	1.025 (1.000, 1.050)	
Yes	16410 (67.77)	1.010 (0.988, 1.032)	1.029 (1.007, 1.051)	
ART methods				0.025
IVF	21367 (67.68)	1.004 (0.985, 1.022)	1.018 (0.999, 1.037)	
ICSI	4833 (65.43)	1.028 (0.988, 1.069)	1.034 (0.992, 1.077)	
IVF+ICSI	2467 (67.72)	1.065 (1.008, 1.126)	1.083 (1.024, 1.146)	
PGT-A	1872 (65.99)	1.024 (0.960, 1.092)	1.073 (1.003, 1.149)	

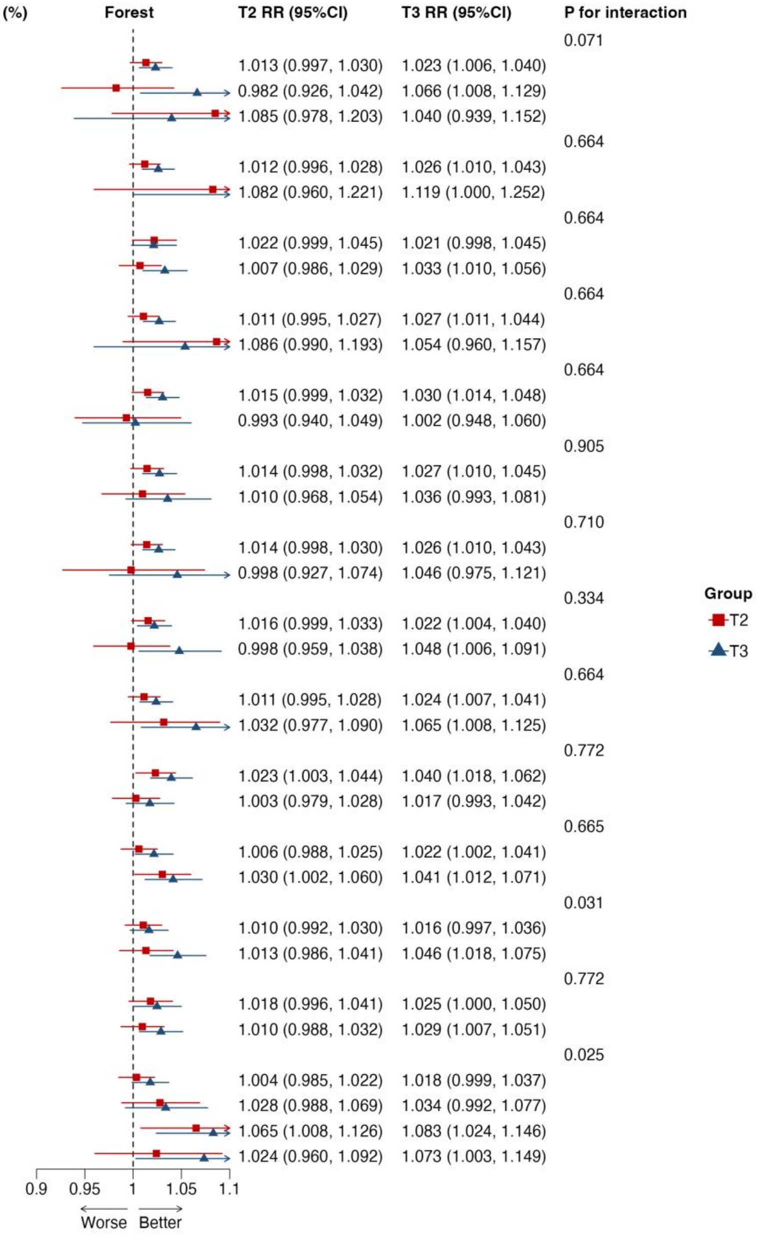

Figure S3. Subgroup group analysis for Clinical Pregnancy in the first transfer cycle of participants by tertiles of TyG-BMI.

Note: Multivariate modified Poisson regression was used to estimate the risk ratios (RRs), the adjusted factors included female age, education level, smoking, alcoholism, clinical or subclinical hypothyroidism, antimullerian hormone, pre-pregnancy hypertension, pre-pregnancy diabetes, gravidity, parity and PCOS. Diabetes drug use means the use of metformin and pioglitazone. T1 group serves as the reference group.

Abbreviations: PCOS = polycystic ovary syndrome; T = tertiles (T1: ⩽177; T2: (177, 203]; T3: >203); CI = confidence interval; RR = risk ratio; Ref = reference abbreviations; TyG-BMI = triglyceride glucose-body mass index.

Subgroup	Live Birth (%)	Forest	T2 RR (95%CI)	T3 RR (95%CI)	P for interaction
Female age					0.044
<35	22287 (60.88)		1.000 (0.981 , 1.020)	0.990 (0.970 , 1.011)	
[35,38)	2551 (49.36)		0.959 (0.893 , 1.030)	0.997 (0.929 , 1.070)	
≥38	1205 (32.91)		1.032 (0.906 , 1.177)	0.904 (0.793 , 1.031)	
Smoking					0.424
No	25494 (57.32)		0.997 (0.978 , 1.016)	0.985 (0.965 , 1.004)	
Yes	549 (56.89)		1.148 (0.989 , 1.333)	1.129 (0.979 , 1.302)	
Sterility type					0.490
primary	11793 (60.87)		1.010 (0.983 , 1.037)	0.980 (0.953 , 1.008)	
Secondary	14250 (54.68)		0.992 (0.966 , 1.018)	0.993 (0.966 , 1.021)	
Clinical or subclinical hypothyroidism					0.490
No	25174 (57.40)		0.998 (0.979 , 1.017)	0.988 (0.969 , 1.008)	
Yes	869 (55.00)		1.048 (0.94 , 1.168)	0.970 (0.868 , 1.084)	
Untreated hydrosalpinx					0.871
No	23642 (57.82)		1.001 (0.981 , 1.021)	0.988 (0.968 , 1.008)	
Yes	2401 (52.80)		0.986 (0.922 , 1.054)	0.983 (0.918 , 1.053)	
Leiomyoma					0.470
No	22057 (58.36)		0.999 (0.979 , 1.02)	0.994 (0.973 , 1.015)	
Yes	3986 (52.15)		1.002 (0.951 , 1.055)	0.962 (0.912 , 1.015)	
Adenomyosis					0.696
No	24602 (57.71)		1.002 (0.983 , 1.021)	0.988 (0.968 , 1.008)	
Yes	1441 (51.34)		0.959 (0.876 , 1.051)	0.978 (0.896 , 1.068)	
Uterine adhesions					0.490
No	21755 (58.22)		1.000 (0.980 , 1.021)	0.982 (0.961 , 1.003)	
Yes	4288 (53.15)		0.990 (0.943 , 1.039)	1.003 (0.954 , 1.056)	
Endometriosis					0.696
No	23680 (57.83)		0.998 (0.979 , 1.018)	0.983 (0.963 , 1.004)	
Yes	2363 (52.60)		1.012 (0.946 , 1.082)	1.025 (0.957 , 1.098)	
PCOS					0.696
No	16682 (55.20)		1.010 (0.986 , 1.034)	0.998 (0.973 , 1.023)	
Yes	9361 (61.52)		0.990 (0.960 , 1.021)	0.980 (0.950 , 1.011)	
Male factor					0.490
No	17953 (56.69)		0.992 (0.97 , 1.015)	0.975 (0.952 , 0.999)	
Yes	8090 (58.74)		1.017 (0.983 , 1.052)	1.011 (0.977 , 1.047)	
Type of embryo transfer cycle					0.131
Fresh	16941 (59.14)		0.993 (0.971 , 1.016)	0.975 (0.952 , 0.999)	
Frozen	9102 (54.21)		1.005 (0.972 , 1.039)	1.006 (0.972 , 1.041)	
Diabetes drug use					0.490
No	12085 (56.94)		0.999 (0.973 , 1.026)	0.973 (0.944 , 1.002)	
Yes	13958 (57.64)		1.002 (0.975 , 1.029)	0.998 (0.972 , 1.025)	
ART methods					0.490
IVF	18168 (57.55)		0.992 (0.970 , 1.014)	0.981 (0.959 , 1.004)	
ICSI	4173 (56.49)		1.016 (0.970 , 1.065)	1.010 (0.961 , 1.062)	
IVF+ICSI	2124 (58.30)		1.036 (0.970 , 1.107)	1.040 (0.972 , 1.114)	
PGT-A	1578 (55.62)		0.999 (0.926 , 1.079)	0.942 (0.863 , 1.029)	

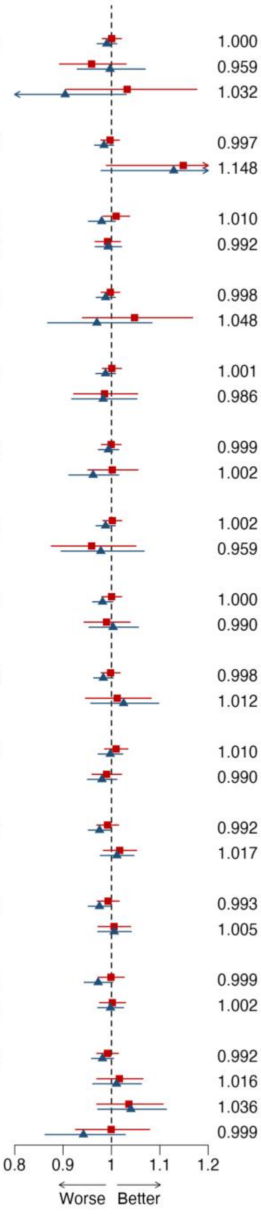

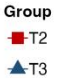

Figure S4. Subgroup group analysis for Live Birth in the first transfer cycle of participants by tertiles of TyG-BMI.

Note: Multivariate modified Poisson regression was used to estimate the risk ratios (RRs), the adjusted factors included female age, education level, smoking, alcoholism, clinical or subclinical hypothyroidism, antimullerian hormone, pre-pregnancy hypertension, pre-pregnancy diabetes, gravidity, parity and PCOS. T1 group serves as the reference group.

Abbreviations: PCOS = polycystic ovary syndrome; T = tertiles (T1: ⩽177; T2: (177, 203]; T3: >203); CI = confidence interval; RR = risk ratio; Ref = reference abbreviations; TyG-BMI = triglyceride glucose-body mass index.

Subgroup	First trimester miscarriage (%)	Forest	T2 RR (95%CI)	T3 RR (95%CI)	P for interaction
Female age					0.789
<35	2295 (8.92)		1.012 (0.920 , 1.114)	1.080 (0.979 , 1.190)	
[35,38)	416 (13.34)		1.169 (0.914 , 1.496)	1.190 (0.934 , 1.515)	
≥38	398 (23.38)		1.123 (0.839 , 1.504)	1.318 (0.996 , 1.744)	
Smoking					0.426
No	3044 (10.18)		1.041 (0.956 , 1.134)	1.128 (1.036 , 1.228)	
Yes	65 (9.98)		0.647 (0.353 , 1.184)	0.546 (0.301 , 0.989)	
Sterility type					0.727
Primary	1172 (8.61)		0.954 (0.831 , 1.095)	1.096 (0.958 , 1.254)	
Secondary	1937 (11.44)		1.084 (0.973 , 1.207)	1.127 (1.011 , 1.256)	
Clinical or subclinical hypothyroidism					0.849
No	3019 (10.22)		1.026 (0.942 , 1.118)	1.111 (1.020 , 1.211)	
Yes	90 (8.90)		1.296 (0.745 , 2.252)	1.203 (0.695 , 2.084)	
Untreated hydrosalpinx					0.890
No	2790 (10.07)		1.032 (0.944 , 1.129)	1.117 (1.022 , 1.221)	
Yes	319 (11.22)		1.016 (0.782 , 1.320)	1.048 (0.804 , 1.367)	
Leiomyoma					0.426
No	2490 (9.69)		1.034 (0.942 , 1.135)	1.071 (0.974 , 1.177)	
Yes	619 (12.81)		1.033 (0.841 , 1.269)	1.263 (1.038 , 1.536)	
Adenomyosis					0.838
No	2874 (9.99)		1.021 (0.935 , 1.114)	1.113 (1.019 , 1.214)	
Yes	235 (13.22)		1.194 (0.857 , 1.665)	1.115 (0.810 , 1.535)	
Uterine adhesions					0.989
No	2544 (10.01)		1.029 (0.937 , 1.131)	1.110 (1.011 , 1.219)	
Yes	565 (11.05)		1.048 (0.865 , 1.270)	1.132 (0.930 , 1.377)	
Endometriosis					0.838
No	2754 (9.95)		1.029 (0.941 , 1.126)	1.128 (1.031 , 1.234)	
Yes	355 (12.44)		1.054 (0.821 , 1.354)	1.000 (0.779 , 1.284)	
PCOS					0.426
No	2091 (10.71)		1.081 (0.975 , 1.200)	1.157 (1.042 , 1.285)	
Yes	1018 (9.24)		0.928 (0.800 , 1.075)	1.006 (0.871 , 1.162)	
Male factor					0.526
No	2210 (10.43)		0.990 (0.895 , 1.094)	1.115 (1.010 , 1.232)	
Yes	899 (9.62)		1.148 (0.981 , 1.344)	1.117 (0.953 , 1.310)	
Type of embryo transfer cycle					0.595
Fresh	1858 (9.44)		1.071 (0.959 , 1.197)	1.165 (1.044 , 1.301)	
Frozen	1251 (11.53)		0.993 (0.871 , 1.132)	1.044 (0.915 , 1.190)	
Diabetes drug use					0.890
No	1444 (10.22)		1.049 (0.932 , 1.181)	1.148 (1.014 , 1.300)	
Yes	1665 (10.15)		1.013 (0.896 , 1.144)	1.081 (0.961 , 1.217)	
ART methods					0.789
IVF	2187 (10.24)		0.988 (0.893 , 1.093)	1.061 (0.959 , 1.173)	
ICSI	468 (9.68)		1.104 (0.892 , 1.365)	1.090 (0.876 , 1.357)	
IVF+ICSI	246 (9.97)		1.194 (0.883 , 1.615)	1.139 (0.837 , 1.551)	
PGT-A	208 (11.11)		1.167 (0.828 , 1.646)	1.787 (1.274 , 2.507)	

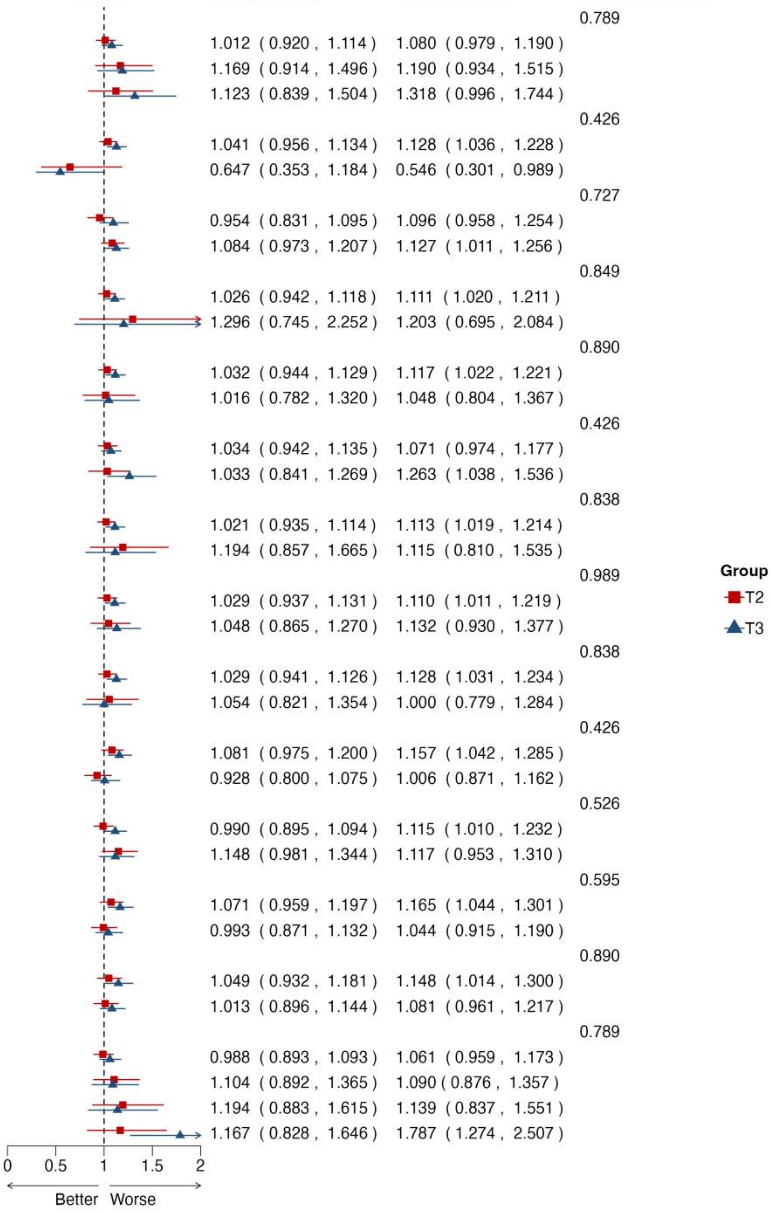

Figure S5. Subgroup group analysis for First trimester miscarriage in the first transfer cycle of participants by tertiles of TyG-BMI.

Note: Multivariate modified Poisson regression was used to estimate the risk ratios (RRs), the adjusted factors included female age, education level, smoking, alcoholism, clinical or subclinical hypothyroidism, antimullerian hormone, pre-pregnancy hypertension, pre-pregnancy diabetes, gravidity, parity and PCOS. T1 group serves as the reference group.

Abbreviations: PCOS = polycystic ovary syndrome; T = tertiles (T1: ⩽177; T2: (177, 203]; T3: >203); CI = confidence interval; RR = risk ratio; Ref = reference abbreviations; TyG-BMI = triglyceride glucose-body mass index.

Subgroup	2nd or 3rd trimester fetal loss (%)	Forest	T2 RR (95%CI)	T3 RR (95%CI)	P for interaction
Female age					0.648
<35	1111 (4.32)		1.305 (1.125 , 1.514)	1.627 (1.406 , 1.883)	
[35,38)	149 (4.78)		1.011 (0.629 , 1.627)	1.870 (1.220 , 2.867)	
≥38	96 (5.64)		1.426 (0.682 , 2.979)	2.138 (1.058 , 4.320)	
Smoking					0.619
No	1320 (4.42)		1.282 (1.114 , 1.475)	1.667 (1.454 , 1.910)	
Yes	36 (5.53)		0.853 (0.292 , 2.493)	2.245 (0.977 , 5.162)	
Sterility type					0.571
Primary	627 (4.61)		1.465 (1.196 , 1.795)	1.843 (1.512 , 2.246)	
Secondary	729 (4.30)		1.121 (0.927 , 1.356)	1.549 (1.291 , 1.859)	
Clinical or subclinical hypothyroidism					0.463
No	1305 (4.42)		1.273 (1.106 , 1.465)	1.638 (1.429 , 1.878)	
Yes	51 (5.04)		1.305 (0.522 , 3.260)	3.217 (1.451 , 7.133)	
Untreated hydrosalpinx					0.761
No	1237 (4.47)		1.284 (1.109 , 1.485)	1.712 (1.487 , 1.970)	
Yes	119 (4.19)		1.165 (0.735 , 1.848)	1.429 (0.922 , 2.215)	
Leiomyoma					0.619
No	1135 (4.42)		1.289 (1.110 , 1.497)	1.632 (1.411 , 1.888)	
Yes	221 (4.57)		1.181 (0.815 , 1.712)	1.892 (1.339 , 2.674)	
Adenomyosis					0.761
No	1257 (4.37)		1.271 (1.102 , 1.467)	1.648 (1.434 , 1.894)	
Yes	99 (5.57)		1.251 (0.700 , 2.236)	2.034 (1.202 , 3.439)	
Uterine adhesions					0.619
No	1099 (4.32)		1.344 (1.149 , 1.571)	1.746 (1.500 , 2.030)	
Yes	257 (5.03)		1.047 (0.771 , 1.422)	1.526 (1.140 , 2.041)	
Endometriosis					0.761
No	1224 (4.42)		1.271 (1.098 , 1.470)	1.657 (1.439 , 1.907)	
Yes	132 (4.63)		1.296 (0.814 , 2.061)	1.928 (1.244 , 2.990)	
PCOS					
No	729 (3.73)		1.145 (0.947 , 1.383)	1.662 (1.387 , 1.991)	0.600
Yes	627 (5.69)		1.441 (1.174 , 1.768)	1.720 (1.408 , 2.101)	
Male factor					0.463
No	1004 (4.74)		1.398 (1.190 , 1.642)	1.783 (1.521 , 2.089)	
Yes	352 (3.77)		0.957 (0.725 , 1.262)	1.454 (1.131 , 1.868)	
Type of embryo transfer cycle					0.721
Fresh	872 (4.43)		1.286 (1.083 , 1.528)	1.604 (1.356 , 1.897)	
Frozen	484 (4.46)		1.229 (0.971 , 1.555)	1.834 (1.469 , 2.290)	
Diabetes drug use					0.463
No	586 (4.15)		1.420 (1.159 , 1.740)	1.996 (1.632 , 2.441)	
Yes	770 (4.69)		1.136 (0.939 , 1.376)	1.443 (1.203 , 1.731)	
ART methods					0.721
IVF	989 (4.63)		1.333 (1.132 , 1.570)	1.701 (1.451 , 1.993)	
ICSI	188 (3.89)		1.059 (0.745 , 1.505)	1.305 (0.929 , 1.833)	
IVF+ICSI	93 (3.77)		1.141 (0.657 , 1.982)	1.835 (1.079 , 3.119)	
PGT-A	86 (4.59)		1.229 (0.696 , 2.171)	2.282 (1.300 , 4.003)	

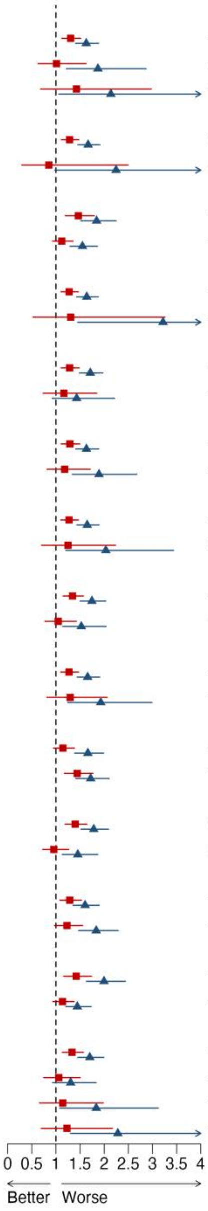

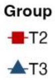

Figure S6. Subgroup group analysis for 2nd or 3rd trimester fetal loss in the first transfer cycle of participants by tertiles of TyG-BMI.

Note: Multivariate modified Poisson regression was used to estimate the risk ratios (RRs), the adjusted factors included female age, education level, smoking, alcoholism, clinical or subclinical hypothyroidism, antimullerian hormone, pre-pregnancy hypertension, pre-pregnancy diabetes, gravidity, parity and PCOS. T1 group serves as the reference group.

Abbreviations: PCOS = polycystic ovary syndrome; T = tertiles (T1: ⩽177; T2: (177, 203]; T3: >203); CI = confidence interval; RR = risk ratio; Ref = reference abbreviations; TyG-BMI = triglyceride glucose-body mass index.

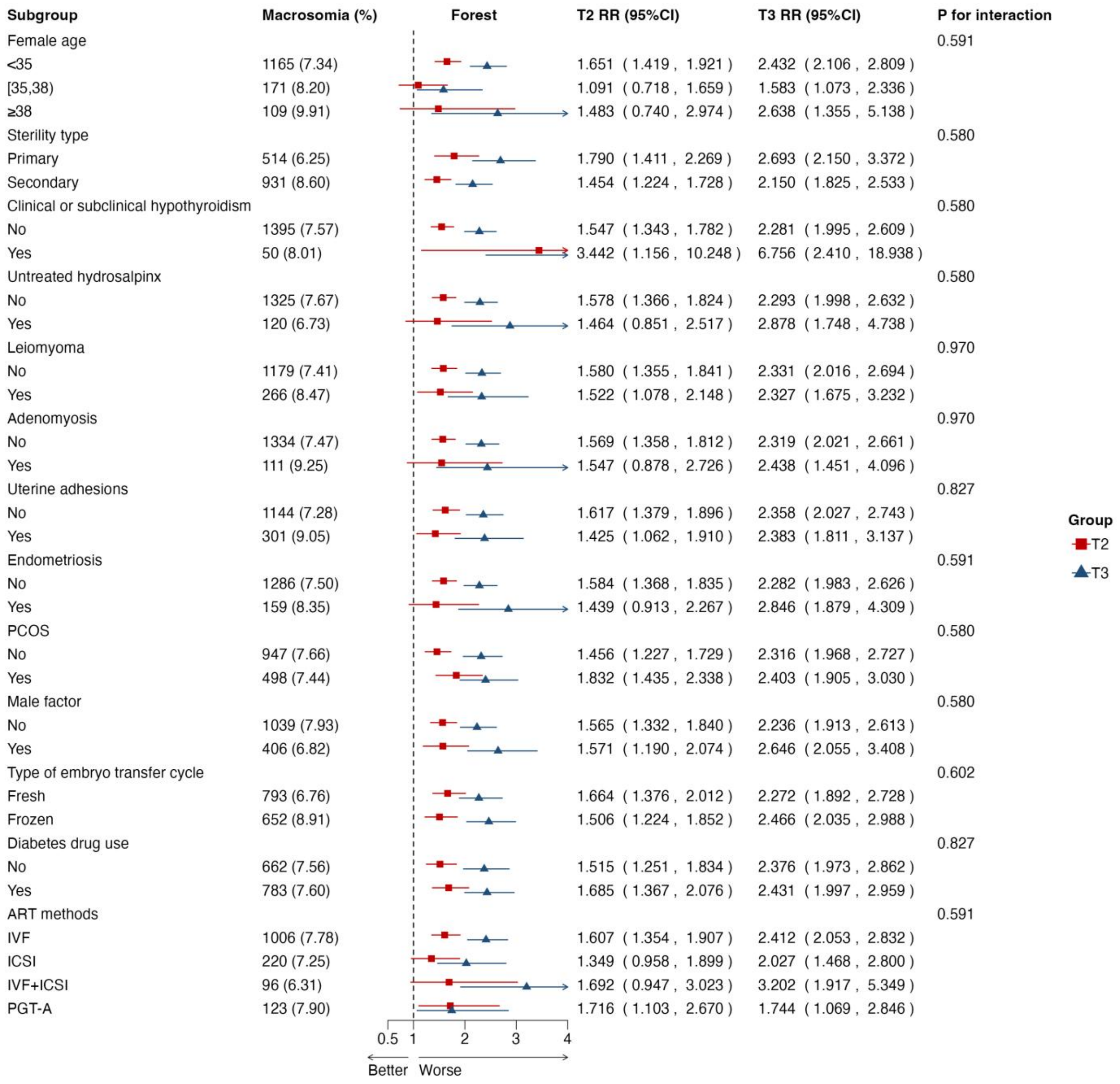

Figure S7. Subgroup group analysis for Macrosomia in the first transfer cycle of participants by tertiles of TyG-BMI.

Note: Multivariate modified Poisson regression was used to estimate the risk ratios (RRs), the adjusted factors included female age, education level, smoking, alcoholism, clinical or subclinical hypothyroidism, antimullerian hormone, pre-pregnancy hypertension, pre-pregnancy diabetes, gravidity, parity and PCOS. T1 group serves as the reference group.

Abbreviations: PCOS = polycystic ovary syndrome; T = tertiles (T1: ⩽177; T2: (177, 203]; T3: >203); CI = confidence interval; RR = risk ratio; Ref = reference abbreviations; TyG-BMI = triglyceride glucose-body mass index.

Subgroup	Preterm Birth (%)	Forest	T2 RR (95%CI)	T3 RR (95%CI)	P for interaction
Female age					0.712
<35	4523 (20.29)		1.097 (1.028 , 1.170)	1.237 (1.160 , 1.319)	
[35,38)	451 (17.68)		1.114 (0.887 , 1.400)	1.153 (0.920 , 1.445)	
≥38	161 (13.36)		1.505 (0.901 , 2.514)	1.789 (1.080 , 2.965)	
Smoking					0.745
No	5012 (19.66)		1.106 (1.039 , 1.177)	1.237 (1.162 , 1.316)	
Yes	123 (22.40)		0.930 (0.603 , 1.436)	1.097 (0.740 , 1.629)	
Sterility type					0.447
Primary	2509 (21.28)		1.063 (0.974 , 1.159)	1.193 (1.095 , 1.299)	
Secondary	2626 (18.43)		1.144 (1.048 , 1.249)	1.280 (1.172 , 1.397)	
Clinical or subclinical hypothyroidism					0.712
No	4953 (19.68)		1.102 (1.035 , 1.174)	1.246 (1.171 , 1.327)	
Yes	182 (20.94)		1.026 (0.738 , 1.426)	0.949 (0.677 , 1.329)	
Untreated hydrosalpinx					0.233
No	4685 (19.82)		1.079 (1.012 , 1.151)	1.214 (1.139 , 1.294)	
Yes	450 (18.74)		1.395 (1.119 , 1.738)	1.508 (1.213 , 1.874)	
Leiomyoma					0.786
No	4404 (19.97)		1.100 (1.029 , 1.176)	1.245 (1.166 , 1.330)	
Yes	731 (18.34)		1.122 (0.948 , 1.329)	1.181 (0.995 , 1.402)	
Adenomyosis					0.712
No	4906 (19.94)		1.096 (1.030 , 1.168)	1.241 (1.166 , 1.321)	
Yes	229 (15.89)		1.287 (0.936 , 1.768)	1.249 (0.909 , 1.716)	
Uterine adhesions					0.197
No	4358 (20.03)		1.100 (1.029 , 1.176)	1.198 (1.120 , 1.280)	
Yes	777 (18.12)		1.106 (0.941 , 1.300)	1.468 (1.255 , 1.716)	
Endometriosis					0.745
No	4767 (20.13)		1.095 (1.028 , 1.167)	1.229 (1.154 , 1.310)	
Yes	368 (15.57)		1.204 (0.946 , 1.532)	1.350 (1.060 , 1.718)	
PCOS					0.447
No	3097 (18.56)		1.140 (1.052 , 1.234)	1.289 (1.190 , 1.398)	
Yes	2038 (21.77)		1.047 (0.949 , 1.154)	1.162 (1.056 , 1.277)	
Male factor					0.871
No	3602 (20.06)		1.097 (1.020 , 1.180)	1.234 (1.147 , 1.327)	
Yes	1533 (18.95)		1.114 (0.992 , 1.250)	1.244 (1.112 , 1.393)	
Type of embryo transfer cycle					0.409
Fresh	3574 (21.10)		1.058 (0.983 , 1.138)	1.180 (1.097 , 1.269)	
Frozen	1561 (17.15)		1.191 (1.062 , 1.334)	1.345 (1.202 , 1.506)	
Diabetes drug use					0.786
No	2346 (19.41)		1.080 (0.990 , 1.179)	1.210 (1.104 , 1.326)	
Yes	2789 (19.98)		1.130 (1.034 , 1.235)	1.269 (1.164 , 1.383)	
ART methods					0.196
IVF	3762 (20.71)		1.106 (1.029 , 1.189)	1.226 (1.141 , 1.316)	
ICSI	802 (19.22)		1.063 (0.913 , 1.237)	1.090 (0.933 , 1.274)	
IVF+ICSI	432 (20.34)		0.938 (0.753 , 1.168)	1.276 (1.038 , 1.570)	
PGT-A	139 (8.81)		1.822 (1.192 , 2.785)	1.994 (1.258 , 3.159)	

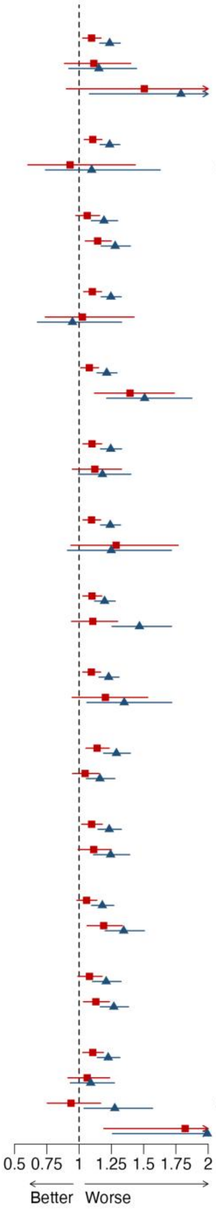

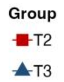

Figure S8. Subgroup group analysis for Preterm Birth in the first transfer cycle of participants by tertiles of TyG-BMI.

Note: Multivariate modified Poisson regression was used to estimate the risk ratios (RRs), the adjusted factors included female age, education level, smoking, alcoholism, clinical or subclinical hypothyroidism, antimullerian hormone, pre-pregnancy hypertension, pre-pregnancy diabetes, gravidity, parity and PCOS. T1 group serves as the reference group.

Abbreviations: PCOS = polycystic ovary syndrome; T = tertiles (T1: ⩽177; T2: (177, 203]; T3: >203); CI = confidence interval; RR = risk ratio; Ref = reference abbreviations; TyG-BMI = triglyceride glucose-body mass index.
